# Supplementary material for: Estimating the costs of blindness and moderate to severe visual impairment among people with diabetes in India
Source: BMJ Open. 2023 Jun 23;13(6):e063390. doi: 10.1136/bmjopen-2022-063390 (PMC10314483; doi:10.1136/bmjopen-2022-063390)
Supplement: Supplementary data [file bmjopen-2022-063390supp001.pdf]

Derivation of the QALY loss associated with blindness and moderate to severe visual impairment (MSVI)

The SMART dataset has responses to questions about sight:

1. I have no problems seeing
2. I have slight problems seeing
3. I have some problems seeing
4. I have severe problems seeing
5. I am unable to see

We equate categories 4 and 5 with blindness and category 3 with MSVI. The comparator is category 1.
The dataset also has responses to the EQ-5D-5L descriptors:

Table 1: Loss of QALYs from five levels of five categories

Level	Mobility	Self Care	Usual Activities	Pain and discomfort	Anxiety and depression
1	I have no problems in walking about	I have no problems washing or dressing myself	I have no problems with performing my usual activities	I have no pain or discomfort	I am not anxious or depressed
2	I have slight problems	I have mild problems	I have mild problems	I have mild pain or discomfort	I am mildly anxious or depressed
3	I have moderate problems	I have moderate problems	I have moderate problems	I have moderate pain or discomfort	I am moderately anxious or depressed
4	I have severe problems	I have severe problems	I have severe problems	I have severe pain or discomfort	I am severely anxious or depressed
5	I am unable to walk about	I am unable to wash or dress myself	I am unable to perform my usual activities	I have extreme pain or discomfort	I am extremely anxious or depressed

The Chinese value set enables the quality adjusted life-year (QALY) value to be derived for a respondent reporting any combination of these levels.

The value set is as follows:*

Table 2: EQ-5D-5L: loss of QALYs from five levels of five forms of health related quality of life					
Level	Mobility	Self Care	Usual Activities	Pain and discomfort	Anxiety and Depression
1	-	-	-	-	-
2	0.066	0.048	0.045	0.058	0.049
3	0.158	0.116	0.107	0.138	0.118
4	0.287	0.210	0.194	0.252	0.215
5	0.345	0.253	0.233	0.302	0.258

For example, a respondent registering level 2 on mobility, 3 on self care, 1 on usual activities, 2 on pain and discomfort and 5 on anxiety and depression would be assigned a QALY level of

1 - 0.066 - 0.116 - 0 - 0.138 - 0.258 = 0.422

We can now estimate the mean age-adjusted QALY loss with blindness and MSVI.

*Luo N, Liu G, Li M, Guan H, Jin X, Rand-Hendriksen K. Estimating an EQ-5D-5L value set for China. Value Health. 2017 Apr;20(4):662-669. doi: 10.1016/j.jval.2016.11.016. Epub 2017 Feb 9. Table 4 column 3.

Table 3: QALY loss with blindness and MSVI						
		Mean QALY by age			Mean QALY by age	
Age (1)	Numbers blind (2)	Blind (3)	No problems seeing (4)	Numbers MSVI (5)	MSVI (6)	No problems seeing (7)
40-44	2	0.340	0.980	10	0.805	As in column (4)
45-49	9	0.590	0.975	25	0.670	
50-54	9	0.550	0.966	54	0.711	
55-59	11	0.627	0.963	56	0.681	
60-64	15	0.442	0.948	75	0.718	
65-69	15	0.605	0.938	109	0.724	
70-74	18	0.509	0.914	92	0.653	
75-79	8	0.595	0.883	57	0.590	
80-84	10	0.355	0.828	54	0.539	
85-89	2	0.208	0.823	18	0.475	
90-94	3	0.266	0.559	6	0.483	
95-99	1	0.481	0.559	1	0.481	
	103			557		
Age weighted mean (blind or MSVI weights)		0.512	0.907		0.662	0.918

The data in columns 3 and 6 are the tariff values corresponding to the EQ5D responses of those SMART participants who report vision categories 4 and 5 or vision category 3 respectively.

QALY loss with blindness = 0.907 - 0.512 = 0.395
QALY loss with MSVI = 0.918 - 0.662 = 0.256

Sensitivity analysis blind =5 and half of 4 MSVI = 3 and half of 4

Table 3: QALY loss with blindness and MSVI						
		Mean QALY by age			Mean QALY by age	
Age (1)	Numbers blind (2)	Blind (3)	No problems seeing (4)	Numbers MSVI (5)	MSVI (6)	No problems seeing (7)
40-44	1.5	0.252	0.980	10.5	0.795	As in column (4)
45-49	4.5	0.590	0.975	29.5	0.658	
50-54	5	0.558	0.966	58	0.699	
55-59	5.5	0.627	0.963	61.5	0.676	
60-64	8	0.429	0.948	82	0.695	
65-69	8	0.576	0.938	116	0.718	
70-74	9.5	0.485	0.914	100.5	0.643	
75-79	4	0.595	0.883	61	0.591	
80-84	5.5	0.305	0.828	58.5	0.529	
85-89	1	0.208	0.823	19	0.461	
90-94	1.5	0.266	0.559	7.5	0.440	
95-99	0.5	0.481	0.559	1.5	0.481	
	54.5			605.5		
Age weighted mean (blind or MSVI weights)		0.492	0.913		0.652	0.918

QALY loss with blindness = 0.913 - 0.492 = 0.421
QALY loss with MSVI = 0.918 - 0.652 = 0.266

Sensitivity analysis

Blind = 4 and 5; MSVI = 3; Comparator = 1 and 2

Table 5: QALY loss with blindness and MSVI						
		Mean QALY by age			Mean QALY by age	
Age (1)	Numbers blind (2)	Blind (3)	No problems seeing (4)	Numbers MSVI (5)	MSVI (6)	No problems seeing (7)
40-44	2	0.340	0.968	10	0.805	As in column (4)
45-49	9	0.590	0.962	25	0.670	
50-54	9	0.550	0.950	54	0.711	
55-59	11	0.627	0.938	56	0.681	
60-64	15	0.442	0.914	75	0.718	
65-69	15	0.605	0.899	109	0.724	
70-74	18	0.509	0.862	92	0.653	
75-79	8	0.595	0.832	57	0.590	
80-84	10	0.355	0.784	54	0.539	
85-89	2	0.208	0.766	18	0.475	
90-94	3	0.266	0.678	6	0.483	
95-99	1	0.481	0.639	1	0.481	
	103			557		
Age weighted mean (blind or MSVI weights)		0.512	0.882		0.662	0.883

QALY loss with blindness = 0.882 - 0.512 = 0.370

QALY loss with MSVI = 0.883 - 0.662 = 0.221
