# Supplementary material for: Estimating the costs of blindness and moderate to severe visual impairment among people with diabetes in India
Source: BMJ Open. 2023 Jun 23;13(6):e063390. doi: 10.1136/bmjopen-2022-063390 (PMC10314483; doi:10.1136/bmjopen-2022-063390)
Supplement: Author's manuscript [file bmjopen-2022-063390.draft_revisions.pdf]

BMJ Open is committed to open peer review. As part of this commitment we make the peer review history of every article we publish publicly available.

When an article is published we post the peer reviewers' comments and the authors' responses online. We also post the versions of the paper that were used during peer review. These are the versions that the peer review comments apply to.

The versions of the paper that follow are the versions that were submitted during the peer review process. They are not the versions of record or the final published versions. They should not be cited or distributed as the published version of this manuscript.

BMJ Open is an open access journal and the full, final, typeset and author-corrected version of record of the manuscript is available on our site with no access controls, subscription charges or pay-per-view fees (http://bmjopen.bmj.com).

If you have any questions on BMJ Open's open peer review process please email info.bmjopen@bmj.com

BMJ Open

ESTIMATING THE COSTS OF BLINDNESS AND MODERATE TO SEVERE VISUAL IMPAIRMENT AMONGST PEOPLE WITH DIABETES IN INDIA

Journal:	*BMJ Open*
Manuscript ID	bmjopen-2022-063390
Article Type:	Original research
Date Submitted by the Author:	29-Mar-2022

Complete List of Authors:	Redding, Stuart; University of Oxford Nuffield Department of Primary Care Health Sciences, Centre for Health Service Economics & Organisation<br>Anderson, Robert; University of Oxford Nuffield Department of Primary Care Health Sciences, Centre for Health Service Economics & Organisation<br>Raman, Rajiv; Sankara Nethralaya, Shri Bhagwan Mahavir Vitreoretinal services<br>Sivaprasad, Sobha; Moorfields Eye Hospital<br>Wittenberg, Raphael; University of Oxford, Centre for Health Service Economics and Organisation

Keywords:	HEALTH ECONOMICS, OPHTHALMOLOGY, General diabetes < DIABETES & ENDOCRINOLOGY

SCHOLARONE™ Manuscripts

I, the Submitting Author has the right to grant and does grant on behalf of all authors of the Work (as defined in the below author licence), an exclusive licence and/or a non-exclusive licence for contributions from authors who are: i) UK Crown employees; ii) where BMJ has agreed a CC-BY licence shall apply, and/or iii) in accordance with the terms applicable for US Federal Government officers or employees acting as part of their official duties; on a worldwide, perpetual, irrevocable, royalty-free basis to BMJ Publishing Group Ltd ("BMJ") its licensees and where the relevant Journal is co-owned by BMJ to the co-owners of the Journal, to publish the Work in this journal and any other BMJ products and to exploit all rights, as set out in our licence.

The Submitting Author accepts and understands that any supply made under these terms is made by BMJ to the Submitting Author unless you are acting as an employee on behalf of your employer or a postgraduate student of an affiliated institution which is paying any applicable article publishing charge ("APC") for Open Access articles. Where the Submitting Author wishes to make the Work available on an Open Access basis (and intends to pay the relevant APC), the terms of reuse of such Open Access shall be governed by a Creative Commons licence – details of these licences and which Creative Commons licence will apply to this Work are set out in our licence referred to above.

Other than as permitted in any relevant BMJ Author's Self Archiving Policies, I confirm this Work has not been accepted for publication elsewhere, is not being considered for publication elsewhere and does not duplicate material already published. I confirm all authors consent to publication of this Work and authorise the granting of this licence.

For peer review only

ESTIMATING THE COSTS OF BLINDNESS AND MODERATE TO SEVERE VISUAL IMPAIRMENT AMONGST PEOPLE WITH DIABETES IN INDIA

Stuart Redding*[1], Robert Anderson[1], Rajiv Raman[2], Sobha Sivaprasad[3] and Raphael Wittenberg[1],

[1] Centre for Health Service Economics & Organisation (CHSEO), University of Oxford
[2] Shri Bhagwan Mahavir Vitreoretinal services, Sankara Nethralaya, Chennai, India
[3]NIHR Biomedical Research Centre, Moorfields Eye Hospital NHS Foundation Trust, London

* Corresponding author:
Centre for Health Service Economics & Organisation
Radcliffe Primary Care Building
Radcliffe Observatory Quarter
Woodstock Road
Oxford OX2 6GG
Email: stuart.redding@phc.ox.ac.uk

For peer review only

For peer review only - http://bmjopen.bmj.com/site/about/guidelines.xhtml

ESTIMATING THE COSTS OF BLINDNESS AND MODERATE TO SEVERE VISUAL IMPAIRMENT AMONGST PEOPLE WITH DIABETES IN INDIA

Abstract

Objectives: This study provides an estimate of the annual cost of blindness and moderate to severe visual impairment (MSVI) among people with diabetes aged 40 and above in Indian subcontinent in the year 2019.

Design: A cost of illness study

Setting: India

Participants: People with diabetes aged 40 years and above in India in the year 2019.

Primary and secondary outcome measures: We provide estimates for the total costs of screening for most common vision threatening eye conditions, treatment of these conditions, economic activity lost by these people and their family carers whose ability to work is affected, and loss of quality of life experienced by people with diabetes and blindness or MSVI.

Results: We estimate that for people with diabetes aged 40 years or above, annual screening followed by eye examination where required would cost around INR 42.3 billion (4230 crores) per year; treating sight problems around INR 2.865 billion (286.5 crores) per year if 20% of those needing treatment receive it; and lost economic activity between INR 399 million (39.9 crores) and INR 789 million (78.9 crores) annually depending on assumptions and that 2.5 million (0.25 crores) quality-adjusted life years (QALYs) are lost annually due to blindness and MSVI.

Conclusions: We believe that this is the first study to estimate the cost of blindness and MSVI for people aged 40 and over with diabetes in India. The annual cost to the Indian economy is substantial. We would expect his cost to fall if a successful screening and treatment plan is introduced in India. We suggest further work using more robust data, when available, to estimate the loss of productivity and loss of QALYs would be worthwhile.

For peer review only - http://bmjopen.bmj.com/site/about/guidelines.xhtml

'Strengths and limitations of this study'

We believe that this is the first study to estimate the cost of blindness and MSVI for people aged 40 and over with diabetes in India.

We use a range of data including recent data on costs of screening from Kerala, but we cannot be certain that the costs of screening would be similar across other states in India.

We take care to make assumptions, in particular that it would not be realistic for the full backlog of people needing treatment to all be treated in just one year.

We use two alternative sets of assumptions for lost productivity: collection of data would be valuable to enable a more robust estimate to be made of lost productivity.

The data on the quality of life of people with eye conditions is drawn from a recent study in India, but the sample with severe sight problems is fairly small.

1. Introduction

Blindness, defined by the World Health Organisation as Snellen visual acuity of 3/60 or worse in the better eye, is a debilitating condition which, in addition to the disabling consequences for the person affected, generates substantial care and treatment costs for society, and also reduces the potential output of the economy by decreasing productivity – of both those who lose their sight and those who have to stop or reduce work in order to provide unpaid care for people who are blind.

Significant work has been undertaken to measure and reduce the prevalence of blindness in India over the last twenty years. Much of this work has been influenced by or undertaken directly as part of the Vision 2020 initiative implemented by the World Health Organisation (WHO), which aimed to eliminate avoidable blindness by 2020[1], and the WHO Global Action Plan for Universal Eye Health 2014-2019, which set a target of a reduction in the prevalence of visual impairment by 25% by 2019 from the baseline level of 2010. Actions taken have facilitated a 47.1% reduction in the prevalence of blindness, 52.6% in Moderate to Severe Visual Impairment (MSVI) and 51.9% in Visual Impairment (VI) during this time period, mainly by reducing the cataract burden and by correcting refractive errors.[2]

For peer review only - http://bmjopen.bmj.com/site/about/guidelines.xhtml

One of the main preventable causes of blindness is diabetic retinopathy (DR). This is a microvascular complication of diabetes that can cause diabetic macular oedema or proliferative diabetic retinopathy, both of which can result in vision impairment and progress to irreversible sight loss. Together they are termed sight threatening DR (STDR). Among individuals with diabetes, the global prevalence of DR was 22.3% in 2020.[3] As life expectancy continues to increase, the numbers of people with diabetes and complications such as DR are expected to rise unless people adopt healthier lifestyles or preventive interventions become available.[4] In addition, people with diabetes are also affected by other common causes of blindness such as cataract and glaucoma and so rates of blindness in people with diabetes exceeds those of people without diabetes.

In this study we report the findings of a cost of illness study in which we attempt to quantify an annual value of the total economic impact of blindness and treatment of sight-threatening conditions amongst people with diabetes aged 40 years and over in India. Cost of illness studies identify and measure direct, indirect and intangible costs associated with a disease and show the total burden of that disease on society. Our cost of illness study estimates the total costs of screening people aged 40 and over with diabetes, healthcare for those found to have eye conditions and lost productivity by those who are blind and by family and friends providing unpaid care for them. We use data on the prevalence of blindness and MSVI among people with diabetes; service utilisation by those with eye conditions and associated cost data from multiple sources; and economic activity lost due to blindness or MSVI by people with diabetes and by those who stop or reduce work to care for them. We also provide an estimate of the loss in quality of life experienced by people with diabetes who suffer blindness or MSVI.

Monitoring and screening patients with diabetes for DR also identifies other eye conditions that cause visual impairment that require treatment. We therefore include the costs of treatment of cataract and glaucoma as well as of DR amongst people with diabetes presenting with MSVI or blindness.

This cost of illness study is part of the ORNATE-India research project[5] which aims to evaluate cost-effective measures for screening for diabetes and its complications and to examine the potential impact of a reduction in the prevalence of blindness on the Indian economy. The project includes an evaluation of a pilot screening programme for DR in Kerala, the

For peer review only

Nayanamritham project[6,7], and an evaluation of a community screening programme for diabetes and its complications in 20 areas of India, the SMART India project[8].

The direct medical cost of DR alone has been explored in countries such as the USA[9], Hungary[10], and Singapore.[11] There has also been cost-of-illness analysis of Type II diabetes and its complications in lower-middle income countries (LMIC) such as Iran[12]. The economic impact of DR alone has been measured in Germany[13] and the USA,[14] amongst other countries, but, to the best of our knowledge, this is the first attempt to adopt a holistic and pragmatic approach for India incorporating all common causes of blindness and MSVI that can affect people with diabetes.

Studies to measure the economic impact of all-cause blindness in India have been limited. The most recent complete estimate of the economic cost of blindness in India[15] of which we are aware was produced more than twenty years ago. That paper produced an estimate of the burden of blindness in India of INR159 billion for 1997, and a cumulative lifetime loss of INR 2,787 billion. It also estimated that treating all cases of blindness due to cataract would cost INR5.3 billion but did not evaluate the impact of STDR in people with diabetes.

The estimate we provide relates to the annual cost of retinal screening for people aged 40 and over who have diabetes in 2020, identifying and treating all common causes of preventable blindness and MSVI and lost productivity by people with diabetes who are blind or have MSVI and their unpaid carers. This is known as the prevalence approach to cost of illness studies. We do not adopt the incidence approach which would provide an estimate of the lifetime costs for a cohort of people with diabetes related blindness.

The costs of screening and treatment (direct costs) and economic costs of lost productivity (indirect costs) relate to different cohorts. The direct costs relate to people who are referred to hospitals for treatment after being identified during the screening programme as having eye conditions and the indirect costs relate to people who are blind or have MSVI. It is important to understand the overall annual costs incurred by the Indian economy covering both those relating to screening and treatment, including those incurred by patients, and those relating to lost productivity that would be avoided through screening and treatment. Understanding the overall economic consequences of blindness is crucial to ensure that the case for devoting resources to the prevention, treatment and research on eye disease is understood and justified.

For peer review only - http://bmjopen.bmj.com/site/about/guidelines.xhtml

2. Research methods

2.1 Overall Approach

We sought to estimate societal costs of blindness and MSVI amongst people with diabetes aged 40 years and over in India in 2019, including costs of screening and healthcare and of lost economic output for blind people with diabetes, people with MSVI and diabetes and their carers. Results for these three categories of costs are provided separately. All reported estimates are annual costs for India, in INR at 2019 prices (although some of the prevalence data relate to 2020). Cost estimates represent a snapshot for the year and not lifetime costs. Discounting was unnecessary since all costs refer to a 1-year period.

2.2 Data sources

Estimates were derived using data from multiple sources as discussed below.

2.2.1 Number of people affected

We begin by defining the number of people aged 40 and over who have diabetes and people who are 50 and over who have diabetes and blindness or MSVI due to existing eye conditions. Age specific prevalence data for diabetes are drawn from the Global Burden of Disease study (year)[16] and combined with 2020 population data from www.PopulationPyramid.net[17] to provide an estimated aggregate number of people in India aged 40 and over with diabetes in the year 2020. A recent study by the Global Burden of Disease (GBD) Study showed prevalence rates for a number of health conditions for South Asia. As most of the population in South Asia is from India, we consider it appropriate to use these values to estimate the number of cases in India. The age standardised prevalence of blindness in people aged 50 years or above in South Asia is 29.8/1000. Applying this prevalence rate to the estimated number of people aged 50 and over with diabetes shows that there are some 1,225,000 blind people aged 50 and over with diabetes in India.

The Global Burden of Disease 2020 prevalence rates per 1,000 population aged 50 and over for common eye conditions among people with blindness and people with MSVI are set out in Table 1. Also shown are estimates of the number of people (in thousands) aged 50 and over

For peer review only - http://bmjopen.bmj.com/site/about/guidelines.xhtml

with diabetes and each of the eye conditions. These estimates assume that the prevalence of each eye condition except DR is the same for people with diabetes as for the wider population. For DR, which relates only to people with diabetes, the total population aged 50 and over rates in the table imply rates of 3.07 among people with diabetes who are blind and 7.86 among people with diabetes who have MSVI. Summing across the eye conditions, we estimate that there are 1,335,000 people with diabetes aged 50 or above who are blind and 9,096,000 who have MSVI. While there is a difference between the two estimates for number of blind people with diabetes that we have derived from the GBD data - £1.225 million or £1.335 million – we use the higher figure to avoid risk of under-estimation.

Table 1: Prevalence rates per 1,000 population aged 50 and over for common eye conditions among people with blindness and people with MSVI in India

	**Blindness**		**MSVI**	
	**Prevalence**	**Number**	**Prevalence**	**Number**
Age-related macular degeneration	0.92	37,900	3.79	156,100
Cataract	18.4	758,000	84.7	3,489,200
DR	0.472	126,400	1.21	324,000
Glaucoma	1.79	73,700	2.96	121,900
Uncorrected refractive error	3.04	125,200	100.0	4,119,500
Other conditions	5.2	214,200	21.5	885,700
All eye conditions combined		1,335,400		9,096,400

2.2.2 Costing care

Blindness can be treated in some conditions such as cataract but not in others including advanced age-related macular degeneration and diabetic retinopathy. However, there are treatments available to slow the progression and/or prevent blindness and MSVI due to most of these common conditions. We attempt to measure the aggregate costs of these treatments in India. We base the costs of care on evidence from previous literature, expert advice and calculations derived from a pilot scheme run to screen for DR among people with diabetes in Kerala,[8] which was conducted as part of the ORNATE-India research project described in the introduction.

From the Nayanamritham study in Kerala, we assume that the unit cost of initial screening is approximately INR 400, if all people with diabetes in the non-communicable disease (NCD) register are screened. Furthermore, we found that in this study 31% of screened patients were

For peer review only - http://bmjopen.bmj.com/site/about/guidelines.xhtml

referred to hospital services. These may be due to cataract, glaucoma, STDR or ungradable retinal images. We estimated that the unit cost of these referrals was INR 1,150 comprising INR 200 costs of the eye examination and INR 950 travel and lost income costs to the patient and an attendant.

We also consider the cost of treating those who were found through the screening programme to have sight-threatening conditions. The unit costs, including costs to the patient and their family, are estimated as an average across private providers and health insurance coverage. These include INR 15,000 for cataract surgery (data provided by ORNATE research team), INR 5,000 for glaucoma surgery[18] and INR 12,000 for DR treatment (laser or intravitreal injections)[19]. The unit costs and source of our estimates for these interventions are presented in table 2 below:

For peer review only

For peer review only - http://bmjopen.bmj.com/site/about/guidelines.xhtml

Table 2: Unit cost of treatment (INR)

Item	Health care	Travel	Total cost	Source
Screening	400	0	400	Kerala pilot
Eye examination	200	950	1150	Kerala pilot
Glasses	1000		1000	Expert advice
Eye drops	500		500	Assumption
Cataract surgery	15000	950	15950	Expert advice
STDR treatment	12000	2850	14850	Expert advice
Glaucoma surgery	5000	950	5950	John et al 2018
Glaucoma average	1625	238	1862.5	Expert advice
Age related macular degeneration	12000	2850	14850	Expert advice

Cost of blindness estimates for high income countries take into account the cost of low vision rehabilitation, community care and residential care for people suffering from blindness. We have assumed that these services are in their infancy in India and most patients rely on family carers. Therefore, we have not attributed costs to them. In addition, we have not included the cost of hip replacement and depression, which are also included in the costs of blindness in high income countries.

2.2.3 Costing economic losses

The indirect economic costs of blindness and MSVI accrue from loss of output due to reduced productivity of blind or visually impaired people compared to people with full vision, plus the loss of output by carers who leave employment or reduce their working hours to look after family members or friends who are blind.

For peer review only - http://bmjopen.bmj.com/site/about/guidelines.xhtml

Determining an estimate for the aggregate loss of output in India requires use of data relating to hours of work and productivity lost by blind and visually impaired people who could be economically active if they had full sight and hours of work lost by carers who have to cease or reduce work in order to care for blind friends or family members. Some of these data are available and others need to be estimated. Where measures cannot be estimated or calculated, we use the same assumptions as those adopted by Shamanna et al (1998)[15] in their study of all-cause blindness in India. The assumptions that we take from Shamanna are as follows:

a) 80% of blind people are economically inactive
b) the average blind person who is economically active produces 25% of the output achieved by a worker with full sight
c) for each blind person, one economically active worker forgoes 10% of their productive time to care for the blind person.

Furthermore, we assume that people with sight loss over 70 would be economically inactive regardless of their vision. For the purpose of our cost estimate we value full-time equivalent output the Indian average Gross National Income (GNI)) per capita. This was equal to INR147,524 in 2019[20].With these assumptions, economic activity lost (EAL in the equation below) can be calculated as:

EAL = (number blind under 70 - number blind under 70 x 20% x 25% + total number blind x 10%) x (GNP per capita).

We also estimate the economic loss associated with MSVI using our own assumptions for the activity loss associated with MSVI. The assumptions we use are:

a) 40% of people with MSVI are economically inactive
b) the average person with MSVI who is economically active produces 50% of the output achieved by a worker with full sight
c) for each person with MSVI, one economically active worker forgoes 5% of their productive time to care for the person with MSVI.

We also consider similar assumptions presented by Eckert et al (2015)[21]. In their study, they assume that no blind people are able to work, and that for each blind person one potentially active worker forgoes 10% of their productive time to care for the blind person. Furthermore, they assume that all people with MSVI are able to work, but only produce 70% of what a person

For peer review only - http://bmjopen.bmj.com/site/about/guidelines.xhtml

with full sight produces. They assume that for each person with MSVI one potentially active worker forgoes 5% of their productive time to care for the person with MSVI.

2.2.4 Calculating QALY losses

Ill-health can affect health-related quality of life in many ways. The standard metric for measuring health-related quality of life in cost-utility analyses is the quality adjusted life year (QALY). One QALY is equal to one life year 1 in full health; any ill-health that reduces ability to perform day-to-day functions is assumed to reduce quality of life, such that one life year in less than full health is valued at less than one QALY. The standard QALY metric is now increasingly EQ-5D-5L, replacing EQ-5D-3L. Each respondent indicates on a five-point scale their ability to function in five specific dimensions (mobility, self-care, usual activities, pain/discomfort, and anxiety/depression). Each EQ-5D-5L health state can be assigned a value on a scale or tariff where 1=full health and 0=dead. Since there is currently no value set available for India, we utilise the Chinese value set.[22]

In order to quantify the QALY losses associated with blindness and with MSVI, we use individual-level data on these five dimensions combined with data detailing the state of their sight. We take this data from the SMART study, which was part of the overall ORNATE India study as outlined above. The SMART study collected information on EQ-5D-5L health state and on vision using the following categories:

1. I have no problems seeing
2. I have slight problems seeing
3. I have some problems seeing
4. I have severe problems seeing
5. I am unable to see

For the purpose of our analysis we equate blindness with response categories "I have severe problems seeing" or "I am unable to see" and equate MSVI with the response category "I have some problems seeing". In sensitivity analysis we equate MSVI with "I have some problems seeing" or "I have severe problems seeing". In this variant we effectively assume that some of those in the "I have severe problems seeing" category are blind and some have MSVI.

To estimate the loss of QALYs due to blindness and MSVI we treat the response category "I have no problems seeing" as the counterfactual against which to estimate the loss. In sensitivity

For peer review only - http://bmjopen.bmj.com/site/about/guidelines.xhtml

analyses we use both this category and the “I have slight problems seeing” category. The Chinese value set was applied to the EQ5D data for each person to get a quality of life value for each person and then these values were averaged across the relevant subsample by vision category.

2.2.5 Patient and Public Involvement

Patient and the public were not involved in this study.

3. Results

3.1 Prevalence of blindness amongst people with diabetes

In this study we consider people resident in India aged 40 and over who have diabetes. Based on age-stratified prevalence data from the Global Burden of Disease (GBD) for 2019 and age-stratified population data from PopulationPyramid.net for 2020, we estimate that there were 55.9 million people aged 40 and over and almost 41.2 million people aged 50 and over in India with diabetes in 2020. As indicated above, we estimate using GBD data that of these 41.2 million people aged 40 and over there are some 1.34 million blind people with diabetes and 9.10 people with MSVI and diabetes. The breakdown of these estimates by eye condition causing blindness or MSVI is shown in Table 3.

Table 3: Numbers of people with diabetes in India suffering from blindness by eye conditions

	Number of people (50+)	Aged 50+ crude prevalence (per 1,000 people)
With diabetes in India	41,194,000	153.86
With diabetes and all cause blindness	1,335,000	29.80
With diabetes blind due to cataract	758,000	18.40
With diabetes blind due to glaucoma	74,000	1.79
With diabetes blind due to age related macular degeneration	38,000	0.92
With diabetes blind due to STDR	126,000	0.47
With diabetes blind due to residual causes	214,000	5.20

3.2 Cost of screening and follow-up treatment

For peer review only - http://bmjopen.bmj.com/site/about/guidelines.xhtml

We assume that annual retinal screening will be offered to all 55.9 million people with diabetes aged 40 and over. The annual cost of screening this group would be INR 22.3 billion assuming the cost of retinal screening per person is INR 400 on the basis of the Kerala pilot.

We assume on the basis of the Kerala pilot screening programme that 31% of those screened, around 17.3 million people, would be referred to hospital services for an eye examination. If all of them attended hospital for an eye examination at a cost of INR 1,150 per individual, the cost of eye examinations for this group would be INR 19.9 billion. The estimated total cost of annual screening followed by eye examination where required would therefore be around INR 42.3 billion. This data is presented in table 4 below.

Table 4: Cost of screening for sight problems amongst people with diabetes aged 40+

	**Annual**	**Cost (INR millions)**		
Item	Number (millions)	Health care	Travel	Total cost
Screening	55.87	22,350		22,350
Eye examination	17.32	3,460	16,450	19,920
Total		25,810	16,450	42,270

Note: 1) All people with diabetes aged 40+ are assumed to be screened
2) 31% of those screened are assumed to need eye examination, as per Kerala pilot

We also consider the cost of treating those found to have DR, cataract or glaucoma and the cost of glasses for those with refractive disorders, using the prevalence rates and unit costs of treatment shown above. We do not consider treatment for AMD or for people with other eye conditions. We also focus on people aged 50 and over since the GBD does not present data for people aged 40 to 49. Since the prevalence of blindness and MSVI in this group are likely to be low, the omission of this group is not likely to have much impact on the numbers of people treated.

It seems improbable that all those with prevalent eye conditions could be treated in one year. It is likely to require many years for the backlog to be cleared. We assume that either 10% of those requiring treatment or 20% of them would be treated annually. On the basis of 10% the cost would be 1.43 billion INR per year, including 110 million INR for patient and attendant travel and loss of income (Table 5). On the basis of 20% the cost would be 2.87 billion INR

For peer review only - http://bmjopen.bmj.com/site/about/guidelines.xhtml

per year, including 220 million INR for patient and attendant travel and loss of income (Table 4). Almost 73% of this cost relates to cataract surgery.

Table 5: Cost of treating sight problems amongst diabetics aged 50+ in India, if 20% of those needing treatment are treated each year

**Intervention**	**Annual**	**Cost (INR millions)**		
	Number (thousands)	Health care	Travel	Total cost
Cataract surgery	151.60	2,270	140	2,420
STDR treatment	25.27	300	70	380
Glaucoma surgery	3.69	20	4	20
Glaucoma eye drops	14.75	7	-	7
Glasses	42.84	40	-	40
**Total**	238.15	2,650	220	2,870

3.3 Economic activity lost

We also estimate the value of economic activity lost due to blindness amongst people with diabetes who are blind, following the method described in section 2.2.3.

We provide four estimates, relating to:

a) Economic activity lost due to blindness using Shamanna assumptions
b) Economic activity lost due to MSVI using our own assumptions
c) Economic activity lost due to blindness using Eckert assumptions
d) Economic activity lost due to MSVI using Eckert assumptions

We assume that output is lost only in the case of people aged 50 to 69 and that those over 70 would not work even if they had better vision. We use data on the numbers of people who suffer blindness and MSVI caused by DR, by age, from South-East Asia,[23] since we could not find data by age for all people with diabetes and blindness or MSVI in India. In this data, 29.5% of the blind aged 50+ with DR are over 70 and 32.0% of those with MSVI aged 50+ with DR are over 70. We therefore consider economic activity relating to 941,000 blind people and 6.186 billion people with MSVI. We use average gross national income (GNI) per capita as an indicator of the value of lost production. GNI per capita was INR 147,524 in 2019 (World Bank website).

For peer review only

We estimate that the economic loss due to blindness is INR 146 billion under the Shamanna assumptions or INR 153 billion under the Eckert assumptions and that the economic loss due to MSVI is INR 616 billion under our assumptions or INR 319 billion under the Eckert assumptions (Table 6).

Table 6: Economic loss as a consequence of blindness or MSVI among the population of India aged 50+ with diabetes (INR millions)

	Loss due to blindness (Shamanna assumptions)	Loss due to blindness (Eckert assumptions)	Loss due to MSVI (own assumptions)	Loss due to MSVI (Eckert assumptions)
Lost productivity of blind people aged 50-69 unable to work	111,100	138,900	228,100	0
Lost productivity of blind people aged 50-69 able to work partially	20,800	0	342,200	273,800
Lost productivity of family carer for all blind people	13,900	13,900	45,600	45,600
Total productivity lost	145,800	152,8000	616,000	319,400

3.4 Loss of quality adjusted life years (QALYs)

We estimate that the quality of life (QoL) of a blind person is 0.395 lower than that of a person with no sight problem, after standardising by age since blind people are on average older than those without sight problems. Blindness thus means that for each year a blind person lives they enjoy 0.395 fewer QALYs than a person without sight problems. For 1.34 million blind people with diabetes aged 40+ 0.53 million QALYs are lost per year due to blindness. Similarly we estimate that the QoL of a person with MSVI is 0.256 lower than that of a person with no sight

For peer review only - http://bmjopen.bmj.com/site/about/guidelines.xhtml

problem, after standardising for age. For 9.10 people with MSVI and diabetes aged 40+, 2.33 million QALYs are lost per year due to MSVI. If each QALY is valued at the GNI per capita for India, for example, the value of these 2.86 million lost QALYs would be INR421.4 billion per year.

As explained above, we conducted sensitivity analyses where we equated MSVI with some problems seeing and severe problems seeing categories (instead of just the former of these) and the counterfactual with no problems seeing and slight problems seeing categories (instead of just the former of these). Under these variants, the loss was in the range 2.5 million to 3.0 million QALYs per year.

4 Discussion

In this study we provide estimates of several types of cost that occur as a result of blindness amongst diabetics in India. We estimate that if 100% of eligible people attended, annual screening followed by eye examination where required would cost around INR 42.3 billion per year; treating sight problems amongst people with diabetes INR 2,865 million per year, if 20% of those needing treatment are treated annually; the lost economic activity per year is between INR 470 billion and INR 760 billion depending on the assumptions; and loss of quality of life is around 2.86 million QALYs per year.

4.1 Previous studies

There have been previous studies looking at the economic burden of diabetes, blindness and DR in countries across the world but none to our knowledge that have looked at this issue specifically in India. Also, those studies that we have identified tend to focus on specific aspects of cost which makes comparisons with our study difficult.

Toth et al (2020)[10] find that the direct cost of treating DR in Hungary was US$145.8 million in 2016 and will increase to US$169.0 million by 2045 but did not estimate lost economic activity. Work in Singapore[11] shows that on average, people with DR utilise medical care costing almost four times as much as a similar group of diabetic patients without DR.

The total national cost of diagnosed type 2 diabetes in Iran was estimated at US $3.78 billion in 2009,[12] consisting of US $2.04 billion direct (medical and non-medical) costs and US $1.73 billion indirect costs. Ophthalmic complications made up 14% of this figure and permanent

For peer review only - http://bmjopen.bmj.com/site/about/guidelines.xhtml

disability, of which blindness would be a major proportion, was responsible for US $452.4 million of the indirect costs. Research from Germany[13] estimated that the total societal cost of DR was €3.51 bn for the year 2002.

Shamanna (1998) estimated that the burden of blindness – in terms of lost output – in India for the year 1997 was INR 159 billion (US$ 4.4 billion), which equates to INR 665 billion at 2020 prices[24]. This would be within the range of our estimates; but Shamanna, while not covering MSVI, considered all blind people of all ages and not just blindness among people aged 40+ with diabetes.

There have been several studies of related topics in the US. Schmier (2009)[25] find that Medicare payments were on average $182 higher per year amongst diabetic claimants with non-proliferative retinopathy than those without ($10,613 v $9,981), and $3,825 higher amongst patients with proliferative retinopathy ($13,806).

Lee et al (2008)[14] found that amongst a sample of US employees in 2004, direct and indirect annual costs of treating employees with DR ($18,218 and $3548) were significantly higher than those without DR (direct = $11,898; indirect = $2374).
It was estimated that in 2004[25], the direct medical costs of treating DR amongst Americans aged 40 to 64 were approximately $300 million, although more recent work suggests that this is a very small proportion of the burden caused by diabetes as a whole - the total estimated economic burden of diagnosed diabetes in the US in 2017[26] was $327 billion, which consisted of $237 billion in direct medical costs and $90 billion in reduced productivity.

A paper relevant to this work by Orji et al (2021)[27] analysed the cost of treating people with DR at a tertiary eye care centre in South India. They considered direct costs of the consultation, investigations, intravitreal injections, and vitreoretinal surgeries. Furthermore, they included indirect costs of transportation and boarding-lodging costs (estimates from a nearby hotel). Their median cost was INR 8,214 but includes patients who received a wide spectrum of treatments.

4.2 Strengths and limitations

For peer review only - http://bmjopen.bmj.com/site/about/guidelines.xhtml

For peer review only

The major strength of this paper is that it is, to the best of our knowledge, the first to estimate the costs of screening and treatment, the value of economic activity lost and the value of QALYs lost due to blindness and MSVI amongst people with diabetes in India.

We have been able to utilise data on the costs of treating patients with DR from a recent new pilot scheme that has been operating in Kerala that uses accessible technology to screen diabetic patients and refers them where necessary for follow-up investigation and treatment in hospital. Whilst we use detailed recent data from Kerala, we cannot be certain that the costs of screening would be similar across other states in India.

Our estimate of the value of lost productivity uses recent data on the prevalence of blindness among people with diabetes and of average incomes in India. A significant limitation however is that, in the absence of evidence on the proportion of blind people with diabetes and of people with MSVI and diabetes who are unable to work, we have used the same assumptions as Shamanna (1998), which are now more than twenty years old, and of Eckert et al (2015). We considered using data from the 2011 Census instead of those assumptions, but there was concern from experts we consulted about the plausibility of Census data, as the Census uses different definitions of blindness and sight loss. This is an area that deserves further exploration.

Our estimate relates to the current position. If screening was introduced for all people aged 40+ with diabetes, there would in due course be fewer blind people and people with MSVI with diabetes and both the QALYs lost and the economic activity lost would be lower. On the other hand, the number of people in India with diabetes is expected to grow significantly in the next decades[28], leaving more people at risk of DR.

There is a potential issue with overlap between estimated QALY loss and economic activity loss such that summing both elements would be controversial and does not seem appropriate. Caution is required when adding the estimated cost of screening and treatment to either the estimated QALY loss or economic activity loss since the costs relate to different groups of people with diabetes. The costs of screening relate to people with diabetes whether or not they have sight problems. The costs of treatment relate mainly to people who have MSVI and are at risk of blindness. The costs of lost productivity and lost QALYs relate to people who are blind or have MSVI. In practice the treatment costs that would be incurred annually would be the

For peer review only - http://bmjopen.bmj.com/site/about/guidelines.xhtml

For peer review only

costs of treating those newly diagnosed following screening, which would for at least several years include part of a backlog of people who have had deteriorating sight which could have been detected earlier if there had been screening. We assume that in the early years some 10% to 20% of those needing treatment would be treated each year.

4.3 Implications

The most important findings of this study are that the annual cost to the Indian economy arising from lost productivity due to blindness and MSVI among people with diabetes is substantial, and that the cost of annual screening of people with diabetes for eye conditions and treating those with sight-threating DR, cataract or glaucoma would also be substantial. Over time, however, the costs of treatment are likely to fall, when the backlog of people with diabetes and untreated eye conditions is cleared. This is before considering the impact of demographic change and inflation. Also over time the costs incurred through lost productivity will also decline, as the proportion of people with diabetes who become blind falls due to regular screening and timely treatment of those at risk of losing their sight. A policy to conduct annual screening followed by treatment where required would therefore yield a net benefit to the Indian economy as well as a benefit to people with diabetes at risk of blindness and their families.

Contributorship statement

SR developed the original concept; acquired data; performed analysis; drafted the paper and approved the submitted version.

RA contributed to elements of the original concept; performed analysis; contributed to the drafting of the paper and approved the submitted version.

RR contributed to acquiring cost data and approved the submitted version.

SS advised on the concept; provided expert guidance throughout; acquired data; and approved the submitted version.

RW contributed to the original concept; acquired data; performed analysis; drafted parts of the paper and approved the submitted version.

Competing interests

None reported.

Funding

For peer review only - http://bmjopen.bmj.com/site/about/guidelines.xhtml

This work is part of the ORNATE India project funded by the GCRF UKRI (MR/P207881/1). The research is supported by the NIHR Biomedical Research Centre at Moorfields Eye Hospital NHS Foundation Trust and UCL Institute of Ophthalmology.

Data sharing statement

The data used in this study is available upon request.

Acknowledgement

The authors would like to thank the experts who contributed information relating to the cost of ophthalmology treatment in India.

Ethics approval:

This study does not involve human or animal participants and ethical approval is not required.

References

1 Pizzarello, L., Abiose, A., Ffytche, T., Duerksen, R., Thulasiraj, R., Taylor, H., Faal, H., Rao, G., Kocur, I. and Resnikoff, S., 2004. VISION 2020: The Right to Sight: a global initiative to eliminate avoidable blindness. *Archives of ophthalmology*, *122*(4), pp.615-620.

2 National Blindness and Visual Impairment Survey 2015-2019. Dr Rajendra Prasad Centre for Ophthalmic Sciences, AIIMS, New Delhi.

3 Teo, Z.L., Tham, Y.C., Yu, M., Chee, M.L., Rim, T.H., Cheung, N., Bikbov, M.M., Wang, Y.X., Tang, Y., Lu, Y. and Wong, I.Y., 2021. Global prevalence of diabetic retinopathy and projection of burden through 2045: systematic review and meta-analysis. *Ophthalmology*, *128*(11), pp.1580-1591.

4 Zheng, Y., Ley, S.H. and Hu, F.B., 2018. Global aetiology and epidemiology of type 2 diabetes mellitus and its complications. *Nature reviews endocrinology*, *14*(2), pp.88-98.

5 Sivaprasad, S., Raman, R., Conroy, D., Wittenberg, R., Rajalakshmi, R., Majeed, A., Krishnakumar, S., Prevost, T., Parameswaran, S., Turowski, P. and Maheswari, U., 2020. The ORNATE India Project: United Kingdom–India Research Collaboration to tackle visual impairment due to diabetic retinopathy. *Eye*, *34*(7), pp.1279-1286.

6 Sivaprasad, S., Netuveli, G., Wittenberg, R., Khobragade, R., Sadanandan, R., Gopal, B., Premnazir, L., Conroy, D., Srinath, J., Ramakrishnan, R. and George, S., 2021. Complex interventions to implement a diabetic retinopathy care pathway in the public health system in Kerala: the Nayanamritham study protocol. *BMJ open*, *11*(6), p.e040577.

7 Sivaprasad, S., Sahasranamam, V.I., George, S., Sadanandan, R., Gopal, B., Premnazir, L., Conroy, D., Srinath, J., Ramakrishnan, R., Vijayanand, S.M. and Wittenberg, R., 2021. Burden of Diabetic Retinopathy amongst People with Diabetes Attending Primary Care in Kerala: Nayanamritham Project. *Journal of Clinical Medicine*, *10*(24), p.5903.

For peer review only - http://bmjopen.bmj.com/site/about/guidelines.xhtml

8 Sivaprasad, S., Raman, R., Rajalakshmi, R., Mohan, V., Deepa, M., Das, T., Ramasamy, K., Prevost, A.T., Wittenberg, R., Netuveli, G. and Lingam, G., 2020. Protocol on a multicentre statistical and economic modelling study of risk-based stratified and personalised screening for diabetes and its complications in India (SMART India). *BMJ open*, *10*(12), p.e039657.

9 Rein, D.B., Zhang, P., Wirth, K.E., Lee, P.P., Hoerger, T.J., McCall, N., Klein, R., Tielsch, J.M., Vijan, S. and Saaddine, J., 2006. The economic burden of major adult visual disorders in the United States. *Archives of ophthalmology*, *124*(12), pp.1754-1760.

10 Tóth, G., Limburg, H., Szabó, D., Sándor, G.L., Nagy, Z.Z. and Németh, J., 2021. Rapid assessment of avoidable blindness-based healthcare costs of diabetic retinopathy in Hungary and its projection for the year 2045. *British Journal of Ophthalmology*, *105*(8), pp.1116-1120.

11 Zhang, X., Low, S., Kumari, N., Wang, J., Ang, K., Yeo, D., Yip, C.C., Tavintharan, S., Sum, C.F. and Lim, S.C., 2017. Direct medical cost associated with diabetic retinopathy severity in type 2 diabetes in Singapore. *PloS one*, *12*(7), p.e0180949.

12 Javanbakht, M., Baradaran, H.R., Mashayekhi, A., Haghdoost, A.A., Khamseh, M.E., Kharazmi, E. and Sadeghi, A., 2011. Cost-of-illness analysis of type 2 diabetes mellitus in Iran. *PloS one*, *6*(10), p.e26864.

13 Happich, M., Reitberger, U., Breitscheidel, L., Ulbig, M. and Watkins, J., 2008. The economic burden of diabetic retinopathy in Germany in 2002. *Graefe's Archive for Clinical and Experimental Ophthalmology*, *246*(1), pp.151-159.

14 Lee, L.J., Yu, A.P., Cahill, K.E., Oglesby, A.K., Tang, J., Qiu, Y. and Birnbaum, H.G., 2008. Direct and indirect costs among employees with diabetic retinopathy in the United States. *Current medical research and opinion*, *24*(5), pp.1549-1559.

15 Shamanna, B.R., Dandona, L. and Rao, G.N., 1998. Economic burden of blindness in India. *Indian journal of ophthalmology*, *46*(3), p.169.

16 https://www.statista.com/statistics/995968/india-diabetes-prevalence-among-males-by-age-group/, https://www.statista.com/statistics/996000/india-diabetes-prevalence-among-females-by-age-group/ (accessed October 2021)

17 https://www.populationpyramid.net/india/2020/ (accessed October 2021)

18 John, D. and Parikh, R., 2017. Cost-effectiveness and cost utility of community screening for glaucoma in urban India. *Public Health*, *148*, pp.37-48.

19 Le, H.G., Ehrlich, J.R., Venkatesh, R., Srinivasan, A., Kolli, A., Haripriya, A., Ravindran, R.D., Thulasiraj, R.D., Robin, A.L., Hutton, D.W. and Stein, J.D., 2016. A sustainable model for delivering high-quality, efficient cataract surgery in southern India. *Health Affairs*, *35*(10), pp.1783-1790.

20 https://data.worldbank.org/indicator/NY.GNP.PCAP.CN?locations=IN accessed February 2022.

21 Eckert, K.A., Carter, M.J., Lansingh, V.C., Wilson, D.A., Furtado, J.M., Frick, K.D. and Resnikoff, S., 2015. A simple method for estimating the economic cost of productivity loss due to blindness and moderate to severe visual impairment. *Ophthalmic epidemiology*, *22*(5), pp.349-355.

22 Luo, N., Liu, G., Li, M., Guan, H., Jin, X. and Rand-Hendriksen, K., 2017. Estimating an EQ-5D-5L value set for China. *Value in Health*, *20*(4), pp.662-669.

For peer review only - http://bmjopen.bmj.com/site/about/guidelines.xhtml

23 https://www.iapb.org/learn/vision-atlas/causes-of-vision-loss/diabetic-retinopathy/ accessed March 2022.

24 https://data.worldbank.org/indicator/FP.CPI.TOTL?end=2020&locations=IN&start=1991 accessed December 2021.

25 Schmier, J.K., Covert, D.W., Lau, E.C. and Matthews, G.P., 2009. Medicare expenditures associated with diabetes and diabetic retinopathy. *Retina*, *29*(2), pp.199-206.

26 Care, D., 2018. Economic Costs of Diabetes in the US in 2017. *Diabetes Care*, *41*, p.917.

27 Orji, A., Rani, P.K., Narayanan, R., Sahoo, N.K. and Das, T., 2021. The economic burden of diabetic retinopathy care at a tertiary eye care center in South India. *Indian Journal of Ophthalmology*, *69*(3), p.666.

28 https://www.diabetesatlas.org/data/en/country/93/in.html accessed December 2021.

For peer review only

For peer review only - http://bmjopen.bmj.com/site/about/guidelines.xhtml

BMJ Open

ESTIMATING THE COSTS OF BLINDNESS AND MODERATE TO SEVERE VISUAL IMPAIRMENT AMONGST PEOPLE WITH DIABETES IN INDIA

Journal:	*BMJ Open*
Manuscript ID	bmjopen-2022-063390.R1
Article Type:	Original research
Date Submitted by the Author:	30-Jan-2023

Complete List of Authors:	Redding, Stuart; University of Oxford Nuffield Department of Primary Care Health Sciences, Centre for Health Service Economics & Organisation<br>Anderson, Robert; University of Oxford Nuffield Department of Primary Care Health Sciences, Centre for Health Service Economics & Organisation<br>Raman, Rajiv; Sankara Nethralaya, Shri Bhagwan Mahavir Vitreoretinal services<br>Sivaprasad, Sobha; Moorfields Eye Hospital<br>Wittenberg, Raphael; University of Oxford, Centre for Health Service Economics and Organisation

**Primary Subject Heading**:	Health economics
Secondary Subject Heading:	Ophthalmology, Diabetes and endocrinology
Keywords:	HEALTH ECONOMICS, OPHTHALMOLOGY, General diabetes < DIABETES & ENDOCRINOLOGY

SCHOLARONE™
Manuscripts

I, the Submitting Author has the right to grant and does grant on behalf of all authors of the Work (as defined in the below author licence), an exclusive licence and/or a non-exclusive licence for contributions from authors who are: i) UK Crown employees; ii) where BMJ has agreed a CC-BY licence shall apply, and/or iii) in accordance with the terms applicable for US Federal Government officers or employees acting as part of their official duties; on a worldwide, perpetual, irrevocable, royalty-free basis to BMJ Publishing Group Ltd ("BMJ") its licensees and where the relevant Journal is co-owned by BMJ to the co-owners of the Journal, to publish the Work in this journal and any other BMJ products and to exploit all rights, as set out in our licence.

The Submitting Author accepts and understands that any supply made under these terms is made by BMJ to the Submitting Author unless you are acting as an employee on behalf of your employer or a postgraduate student of an affiliated institution which is paying any applicable article publishing charge ("APC") for Open Access articles. Where the Submitting Author wishes to make the Work available on an Open Access basis (and intends to pay the relevant APC), the terms of reuse of such Open Access shall be governed by a Creative Commons licence – details of these licences and which Creative Commons licence will apply to this Work are set out in our licence referred to above.

Other than as permitted in any relevant BMJ Author's Self Archiving Policies, I confirm this Work has not been accepted for publication elsewhere, is not being considered for publication elsewhere and does not duplicate material already published. I confirm all authors consent to publication of this Work and authorise the granting of this licence.

For peer review only

ESTIMATING THE COSTS OF BLINDNESS AND MODERATE TO SEVERE VISUAL IMPAIRMENT AMONGST PEOPLE WITH DIABETES IN INDIA

Stuart Redding*[1], Robert Anderson[1], Rajiv Raman[2], Sobha Sivaprasad[3] and Raphael Wittenberg[1] on behalf of the ORNATE India project group.[#]

[1] Centre for Health Service Economics & Organisation (CHSEO), University of Oxford
[2] Shri Bhagwan Mahavir Vitreoretinal services, Sankara Nethralaya, Chennai, India
[3]NIHR Biomedical Research Centre, Moorfields Eye Hospital NHS Foundation Trust, London

* Corresponding author:
Centre for Health Service Economics & Organisation
Radcliffe Primary Care Building
Radcliffe Observatory Quarter
Woodstock Road
Oxford OX2 6GG
Email: stuart.redding@phc.ox.ac.uk

For peer review only

ESTIMATING THE COSTS OF BLINDNESS AND MODERATE TO SEVERE VISUAL IMPAIRMENT AMONGST PEOPLE WITH DIABETES IN INDIA

Abstract

Objectives: This study provides an estimate of the annual cost of blindness and moderate to severe visual impairment (MSVI) among people with diabetes aged 40 and above in Indian subcontinent in the year 2019.

Design: A cost of illness study

Setting: India

Participants: People with diabetes aged 40 years and above in India in the year 2019.

Primary and secondary outcome measures: We provide estimates for the total costs of screening for most common vision threatening eye conditions, treatment of these conditions, economic activity lost by these people and their family carers whose ability to work is affected, and loss of quality of life experienced by people with diabetes and blindness or MSVI.

Results: We estimate that for people with diabetes aged 40 years or above, annual screening followed by eye examination where required would cost around INR 42.3 billion (4230 crores) per year; treating sight problems around INR 2.87 billion (287 crores) per year if 20% of those needing treatment receive it; and lost economic activity around INR 472 billion (47200 crores) and that 2.86 million (0.286 crores) quality-adjusted life years (QALYs) are lost annually due to blindness and MSVI. The estimate of lost production is highly sensitive to the proportion of people with MSVI able to work and how their output compares with that of a person with no visual impairment.

Conclusions: We believe that this is the first study to estimate the cost of blindness and MSVI for people aged 40 and over with diabetes in India. The annual cost to the Indian economy is substantial. We would expect this cost to fall if a successful screening and treatment plan is introduced in India. We suggest further work using more robust data, when available, to estimate the loss of productivity and loss of QALYs would be worthwhile.

For peer review only - http://bmjopen.bmj.com/site/about/guidelines.xhtml

‘Strengths and limitations of this study’

We believe that this is the first study to estimate the cost of blindness and MSVI for people aged 40 and over with diabetes in India.

We use a range of data including recent data on costs of screening for diabetic retinopathy in Kerala, but we cannot be certain that the costs of screening would be similar across other states in India.

We take care to make realistic assumptions, in particular that it would not be realistic for the full backlog of people needing treatment to all be treated in just one year.

We examine two alternative sets of assumptions for lost productivity: collection of data would be valuable to enable a more robust estimate to be made of lost productivity.

The data on the quality of life of people with eye conditions is drawn from a recent study in India, but the sample with severe sight problems is fairly small.

1. Introduction

Blindness, defined by the World Health Organisation as Snellen visual acuity of 3/60 or worse in the better eye [1], is a debilitating condition which, in addition to the disabling consequences for the person affected, generates substantial care and treatment costs for society. While some people who suffer from vision loss are able to work, their productivity is likely to be less than that of a person with better vision. and some may drop out of the labour force entirely. Therefore blindness and sight loss reduces the potential output of the economy by decreasing the output of both those who are directly affected and those who have to stop or reduce work in order to provide unpaid care for people who are blind.

Significant work has been undertaken to measure and reduce the prevalence of blindness in India over the last twenty years. Much of this work has been influenced by or undertaken directly as part of the Vision 2020 initiative implemented by the World Health Organisation (WHO), which aimed to eliminate avoidable blindness by 2020 [2], and the WHO Global Action Plan for Universal Eye Health 2014-2019, which set a target of a reduction in the prevalence of visual impairment by 25% by 2019 from the baseline level of 2010. Actions taken have facilitated a 47.1% reduction in the prevalence of blindness, 52.6% in Moderate to

For peer review only

For peer review only - http://bmjopen.bmj.com/site/about/guidelines.xhtml

Severe Visual Impairment (MSVI) and 51.9% in Visual Impairment (VI) during this time period, mainly by reducing the cataract burden and by correcting refractive errors [3].

One of the main preventable causes of blindness is diabetic retinopathy (DR). This is a microvascular complication of diabetes that can cause diabetic macular oedema or proliferative diabetic retinopathy, both of which can result in vision impairment and progress to irreversible sight loss. Together they are termed sight threatening DR (STDR). Among individuals with diabetes, the global prevalence of DR was 22.3% in 2020 [4]. As life expectancy continues to increase, the numbers of people with diabetes and complications such as DR are expected to rise unless people adopt healthier lifestyles or preventive interventions become available [5]. In addition, people with diabetes are also affected by other common causes of blindness such as cataract and glaucoma and so rates of blindness in people with diabetes exceeds those of people without diabetes.

In this study we report the findings of a cost of illness study in which we attempt to quantify an annual value of the total economic impact of blindness and treatment of sight-threatening conditions amongst people with diabetes aged 40 years and over in India. Cost of illness studies identify and measure direct, indirect and intangible costs associated with a disease and show the total burden of that disease on society: see, for example, Larg and Moss (2011) [6] for a summary of the approaches utilised and their limitations. Our cost of illness study estimates the total costs of screening people aged 40 and over with diabetes, healthcare for those found to have eye conditions and lost productivity by those who are blind and by family and friends providing unpaid care for them. We use data on the prevalence of blindness and MSVI among people with diabetes; service utilisation by those with eye conditions and associated cost data from multiple sources; and economic activity lost due to blindness or MSVI by people with diabetes and by those who stop or reduce work to care for them. We also provide an estimate of the loss in quality of life experienced by people with diabetes who suffer blindness or MSVI.

Monitoring and screening patients with diabetes for DR also identifies other eye conditions that cause visual impairment that require treatment. We therefore include the costs of treatment of cataract and glaucoma as well as of DR amongst people with diabetes presenting with MSVI or blindness.

For peer review only - http://bmjopen.bmj.com/site/about/guidelines.xhtml

This cost of illness study is part of the ORNATE-India research project [7] which aims to evaluate cost-effective measures for screening for diabetes and its complications and to examine the potential impact of a reduction in the prevalence of blindness on the Indian economy. The project includes an evaluation of a pilot screening programme for DR in Kerala, the Nayanamritham project [8,9], and an evaluation of a community screening programme for diabetes and its complications in 20 areas of India covering all the six regions, the SMART India project[10].

The direct medical cost of DR alone has been explored in countries such as the USA [11], Hungary [12], and Singapore [13]. There has also been cost-of-illness analysis of Type II diabetes and its complications in lower-middle income countries (LMIC) such as Iran [14]. The economic impact of DR alone has been measured in Germany [15] and the USA [16], amongst other countries, but, to the best of our knowledge, this is the first attempt to adopt a holistic and pragmatic approach for India incorporating all common causes of blindness and MSVI that can affect people with diabetes.

Studies to measure the economic impact of all-cause blindness in India have been limited. The most recent complete estimate of the economic cost of blindness in India [17] of which we are aware was produced more than twenty years ago. That paper produced an estimate of the burden of blindness in India of INR159 billion for 1997, and a cumulative lifetime loss of INR 2,787 billion. It also estimated that treating all cases of blindness due to cataract would cost INR5.3 billion but did not evaluate the impact of STDR in people with diabetes.

The estimate we provide relates to the annual cost of retinal screening for people aged 40 and over who have diabetes in 2020, identifying and treating all common causes of preventable blindness and MSVI and lost productivity by people with diabetes who are blind or have MSVI and their unpaid carers. This is known as the prevalence approach to cost of illness studies. We do not adopt the incidence approach which would provide an estimate of the lifetime costs for a cohort of people with diabetes related blindness.

The costs of screening and treatment (direct costs) and economic costs of lost productivity (indirect costs) relate to different cohorts. The direct costs relate to people who are referred to hospitals for treatment after being identified during the screening programme as having eye conditions and the indirect costs relate to people who are blind or have MSVI. It is important

For peer review only - http://bmjopen.bmj.com/site/about/guidelines.xhtml

to understand the overall annual costs incurred by the Indian economy covering both those relating to screening and treatment, including those incurred by patients, and those relating to lost productivity that would be avoided through screening and treatment. Understanding the overall economic consequences of blindness is crucial to ensure that the case for devoting resources to the prevention, treatment and research on eye disease is understood and justified.

2. Research methods

2.1 Overall Approach

We sought to estimate societal costs of blindness and MSVI amongst people with diabetes aged 40 years and over in India in 2019, including costs of screening and healthcare and of lost economic output for blind people with diabetes, people with MSVI and diabetes and their carers. Results for these three categories of costs are provided separately. All reported estimates are annual costs for India, in INR at 2019 prices (although some of the prevalence data relate to 2020). Cost estimates represent a snapshot for the year and not lifetime costs. Discounting was unnecessary since all costs refer to a 1-year period.

2.2 Data sources

Estimates were derived using data from multiple sources as discussed below.

2.2.1 Number of people affected

We begin by defining the number of people aged 40 and over who have diabetes and people who are 50 and over who have diabetes and blindness or MSVI due to existing eye conditions. Age specific prevalence data for diabetes are drawn from the Global Burden of Disease study [18] and combined with 2020 population data from www.PopulationPyramid.net [19] to provide an estimated aggregate number of people in India aged 40 and over with diabetes in the year 2020. A recent study by the Global Burden of Disease (GBD) Study [20] showed prevalence rates for a number of health conditions for South Asia. As most of the population in South Asia is from India, we consider it appropriate to use these values to estimate the number of cases in India. The age standardised prevalence of blindness in people aged 50 years or above in South Asia is 29.8/1000.

For peer review only - http://bmjopen.bmj.com/site/about/guidelines.xhtml

The Global Burden of Disease 2020 prevalence rates per 1,000 population aged 50 and over for common eye conditions among people with blindness and people with MSVI are set out in Table 1. Also shown are estimates of the number of people (in thousands) aged 50 and over with diabetes and each of the eye conditions. These estimates assume that, with the exception of DR which can only affect people with diabetes, the prevalence of each eye condition is the same for people with diabetes as for the wider population. The prevalence in the table, which relate to total population aged 50 and over, imply DR rates of 3.07 among people with diabetes who are blind and 7.86 among people with diabetes who have MSVI. Summing across the eye conditions, we estimate that there are around 1,335,000 blind people aged 50 and over with diabetes and 9,096,000 people with MSVI and diabetes aged 50 and over.

Table 1: Prevalence rates per 1,000 population aged 50 and over for common eye conditions among people with blindness and people with MSVI in India

	Blindness		MSVI	
	**Prevalence**	**Number of diabetic people with eye condition**	**Prevalence**	**Number of diabetic people with eye condition**
Age-related macular degeneration	0.92	37,900	3.79	156,100
Cataract	18.4	758,000	84.7	3,489,200
DR	0.472	126,400	1.21	324,000
Glaucoma	1.79	73,700	2.96	121,900
Uncorrected refractive error	3.04	125,200	100.0	4,119,500
Other conditions	5.2	214,200	21.5	885,700
All eye conditions combined		1,335,400		9,096,400

2.2.2 Costing care

Blindness can be treated in some conditions such as cataract but not in others including advanced age-related macular degeneration and diabetic retinopathy. However, there are treatments available to slow the progression and/or prevent blindness and MSVI due to most of these common conditions. We attempt to measure the aggregate costs of these treatments in India. We base the costs of care on evidence from previous literature, expert advice and calculations derived from a pilot scheme run to screen for DR among people with diabetes in

For peer review only - http://bmjopen.bmj.com/site/about/guidelines.xhtml

Kerala [10] which was conducted as part of the ORNATE-India research project described in the introduction.

From the Nayanamritham study in Kerala, we assume that the unit cost of initial screening is approximately INR 400, if all people with diabetes in the non-communicable disease (NCD) register are screened. This estimate includes INR 85 staff costs, INR 25 cost of admin support, INR 200 annuitized cost of the cameras and 105 annuitized cost of staff training in the Kerala pilot. Information on staff salaries, staff time per person screened, costs of cameras and costs of staff training were provided by the Kerala team. Further details are reported elsewhere (Wittenberg et al, 2023) [21].

Furthermore, we found that in this study 31% of screened patients were referred to hospital services. These may be due to cataract, glaucoma, STDR or ungradable retinal images. We estimated that the unit cost of these referrals was INR 1,150 comprising INR 200 staff costs of the eye examination and INR 950 travel and lost income costs to the patient and an attendant.

We also consider the cost of treating those who were found through the screening programme to have sight-threatening conditions. The unit costs, including costs to the patient and their family, are estimated as an average across private providers and health insurance coverage. These include INR 15,000 for cataract surgery, INR 5,000 for glaucoma surgery [22] and INR 12,000 for DR treatment (laser or intravitreal injections) [23]. The unit costs and sources of our estimates for these interventions are presented in table 2 below. The expert advice was provided by senior clinicians from the SMART India research project.

For peer review only

For peer review only - http://bmjopen.bmj.com/site/about/guidelines.xhtml

Table 2: Unit cost of treatment (INR)

Item	Health care	Travel	Total cost	Source
Screening	400	0	400	Kerala pilot
Eye examination	200	950	1150	Kerala pilot
Glasses	1000		1000	Expert advice
Eye drops	500		500	Expert advice
Cataract surgery	15000	950	15950	Expert advice
STDR treatment	12000	2850	14850	Expert advice
Glaucoma surgery	5000	950	5950	John et al 2017
Glaucoma average	1625	238	1862.5	Expert advice
Age related macular degeneration	12000	2850	14850	Expert advice

Cost of blindness estimates for high income countries take into account the cost of low vision rehabilitation, community care and residential care for people suffering from blindness. We have assumed that these services are in their infancy in India and most patients rely on family carers. Therefore, we have not attributed costs to them. In addition, we have not included the cost of hip replacement and depression, which are also included in the costs of blindness in high income countries.

2.2.3 Costing economic losses

The indirect economic costs of blindness and MSVI accrue from loss of output due to reduced productivity of blind or visually impaired people compared to people with full vision, plus the loss of output by carers who leave employment or reduce their working hours to look after family members or friends who are blind.

For peer review only

For peer review only - http://bmjopen.bmj.com/site/about/guidelines.xhtml

Determining an estimate for the aggregate loss of output in India requires use of data relating to hours of work and productivity lost by blind and visually impaired people who could be economically active if they had full sight and hours of work lost by carers who have to cease or reduce work in order to care for blind friends or family members. Some of these data are available and others need to be estimated. Where data are not available, we use the same assumptions as those adopted by Eckert et al (2015) [24]. In their study, they assume that no blind people are able to work, and that for each blind person one potentially active worker forgoes 10% of their productive time to care for the blind person. Furthermore, they assume that all people with MSVI are able to work, but only produce 70% of what a person with full sight produces. They assume that for each person with MSVI one potentially active worker forgoes 5% of their productive time to care for the person with MSVI.

Furthermore, we assume that people with sight loss over 70 would be economically inactive regardless of their vision. For the purpose of our cost estimate we value full-time equivalent output per person at the Indian average Gross National Income (GNI)) per capita. This was INR147,524 in 2019 [25]. With these assumptions, economic activity lost (EAL in the equation below) can be calculated as:

EAL (blind) = (number blind under 70 + total number blind x 10%) x (GNP per capita)

EAL (MSVI) = (number MSVI under 70 x 30% + total number MSVI x 5%) x (GNP per capita)

We also present, as a sensitivity analysis, an estimate for blind people based on assumptions presented by Shamanna et al (1998) [17] in their study of all-cause blindness in India. The assumptions that we take from Shamanna are as follows:

a) 80% of blind people are economically inactive
b) the average blind person who is economically active produces 25% of the output achieved by a worker with full sight
c) for each blind person, one economically active worker forgoes 10% of their productive time to care for the blind person.

They do not provide estimates for people with MSVI.

For peer review only - http://bmjopen.bmj.com/site/about/guidelines.xhtml

For peer review only

2.2.4 Calculating QALY losses

Ill-health can affect health-related quality of life in many ways. The standard metric for measuring health-related quality of life in cost-utility analyses is the quality adjusted life year (QALY). One QALY is equal to one life year 1 in full health; any ill-health that reduces ability to perform day-to-day functions is assumed to reduce quality of life, such that one life year in less than full health is valued at less than one QALY. The standard QALY metric is now increasingly EQ-5D-5L, replacing EQ-5D-3L. Each respondent indicates on a five-point scale their ability to function in five specific dimensions (mobility, self-care, usual activities, pain/discomfort, and anxiety/depression). Each EQ-5D-5L health state can be assigned a value on a scale or tariff where 1=full health and 0=dead. Since there is currently no value set available for India, we utilise the Chinese value set [26].

In order to quantify the QALY losses associated with blindness and with MSVI, we use individual-level data on these five dimensions combined with data detailing the state of their sight. We take this data from the SMART study, which was part of the overall ORNATE India study as outlined above. The SMART study collected information on EQ-5D-5L health state and on vision using the following self-reported categories:

1. I have no problems seeing
2. I have slight problems seeing
3. I have some problems seeing
4. I have severe problems seeing
5. I am unable to see

For the purpose of our analysis we equate blindness with response categories "I have severe problems seeing" or "I am unable to see" and equate MSVI with the response category "I have some problems seeing". In sensitivity analysis we continue to equate blindness with "I am unable to see" and MSVI with "I have some problems seeing" but we divide the "I have severe problems seeing" category equally between those who are blind and those who have MSVI.

To estimate the loss of QALYs due to blindness and MSVI we treat the response category "I have no problems seeing" as the counterfactual against which to estimate the loss. In sensitivity analyses we use both this category and the "I have slight problems seeing" category. The

For peer review only - http://bmjopen.bmj.com/site/about/guidelines.xhtml

Chinese value set was applied to the EQ5D data for each person to get a quality of life value for each person and then these values were averaged across the relevant subsample by vision category.

2.2.5 Patient and Public Involvement

Patients and the public were not involved in this study.

3. Results

3.1 Prevalence of blindness amongst people with diabetes

In this study we consider people resident in India aged 40 and over who have diabetes. Based on age-stratified prevalence data from the Global Burden of Disease (GBD) for 2019 and age-stratified population data from PopulationPyramid.net for 2020, we estimate that there were 55.9 million people aged 40 and over and almost 41.2 million people aged 50 and over in India with diabetes in 2020. As indicated above, we estimate using GBD data that of these 41.2 million people aged 40 and over there are some 1.34 million blind people with diabetes and 9.10 million people with MSVI and diabetes. The breakdown of these estimates by eye condition causing blindness or MSVI is shown in Table 3.

For peer review

Table 3: Numbers of people with diabetes in India suffering from blindness by eye conditions

	Number of people (50+)	Aged 50+ crude prevalence (per 1,000 people)
With diabetes in India	41,194,000	153.86
With diabetes and all cause blindness	1,335,000	29.80
With diabetes blind due to cataract	758,000	18.40
With diabetes blind due to glaucoma	74,000	1.79
With diabetes blind due to age related macular degeneration	38,000	0.92
With diabetes blind due to STDR	126,000	0.47
With diabetes blind due to residual causes	214,000	5.20

3.2 Cost of screening and follow-up treatment

For peer review only - http://bmjopen.bmj.com/site/about/guidelines.xhtml

We assume that annual retinal screening will be offered to all 55.9 million people with diabetes aged 40 and over. The annual cost of screening this group would be INR 22.3 billion assuming the cost of retinal screening per person is INR 400 on the basis of the Kerala pilot.

We assume on the basis of the Kerala pilot screening programme that 31% of those screened, around 17.3 million people, would be referred to hospital services for an eye examination. If all of them attended hospital for an eye examination at a cost of INR 1,150 per individual, the cost of eye examinations for this group would be INR 19.9 billion. The estimated total cost of annual screening followed by eye examination where required would therefore be around INR 42.3 billion. This data is presented in table 4 below.

Table 4: Cost of screening for sight problems amongst people with diabetes aged 40+

	Annual	Cost (INR millions)		
Item	Number (millions)	Health care	Travel	Total cost
Screening	55.87	22,350		22,350
Eye examination	17.32	3,460	16,450	19,920
Total		25,810	16,450	42,270

Note: 1) All people with diabetes aged 40+ are assumed to be screened

2) 31% of those screened are assumed to need eye examination, as per Kerala pilot

We also consider the cost of treating those found to have DR, cataract or glaucoma and the cost of glasses for those with refractive disorders, using the prevalence rates and unit costs of treatment shown above. We do not consider treatment for AMD or for people with other eye conditions. We also focus on people aged 50 and over since the GBD does not present data for people aged 40 to 49. Since the prevalence of blindness and MSVI in this group are likely to be low, the omission of this group is not likely to have much impact on the numbers of people treated.

It seems improbable that all those with prevalent eye conditions could be treated in one year. It is likely to require many years for the backlog to be cleared. We assume that either 10% of those requiring treatment or 20% of them would be treated annually. On the basis of 10% the cost would be 1.43 billion INR per year, including 110 million INR for patient and attendant travel and loss of income. On the basis of 20% the cost would be 2.87 billion INR per year,

For peer review only - http://bmjopen.bmj.com/site/about/guidelines.xhtml

For peer review only

including 220 million INR for patient and attendant travel and loss of income (Table 5). Almost 73% of this cost relates to cataract surgery.

Table 5: Cost of treating sight problems amongst diabetics aged 50+ in India, if 20% of those needing treatment are treated each year

**Intervention**	**Annual**	**Cost (INR millions)**		
	Number (thousands)	Health care	Travel	Total cost
Cataract surgery	151.60	2,270	140	2,420
STDR treatment	25.27	300	70	380
Glaucoma surgery	3.69	20	4	20
Glaucoma eye drops	14.75	7	-	7
Glasses	42.84	40	-	40
**Total**	238.15	2,650	220	2,870

3.3 Economic activity lost

We also estimate the value of economic activity lost due to blindness amongst people with diabetes who are blind, following the method described in section 2.2.3.

We provide estimates, relating to:

a) Economic activity lost due to blindness using Eckert et al (2015) [24] assumptions
b) Economic activity lost due to blindness using Shamanna et al (1998) [17] assumptions
c) Economic activity lost due to MSVI using Eckert assumptions

We assume that output is lost only in the case of people aged 50 to 69 and that those over 70 would not work even if they had better vision. We use data on the numbers of people who suffer blindness and MSVI caused by DR, by age, from South-East Asia [26], since we could not find data by age for all people with diabetes and blindness or MSVI in India. In this data, 29.5% blind people aged 50+ with DR are over 70 and 32.0% of those with MSVI aged 50+ with DR are over 70. We therefore consider economic activity relating to 941,000 blind people and 6.186 billion people with MSVI. We use average gross national income (GNI) per capita as an indicator of the value of lost production. GNI per capita was INR 147,524 in 2019 [25].

For peer review only

We estimate that the economic loss due to blindness is INR 153 billion under the Eckert assumptions or INR 146 billion under the Shamanna assumptions, and that the economic loss due to MSVI is INR 319 billion under the Eckert assumptions (Table 6).

Table 6: Economic loss as a consequence of blindness or MSVI among the population of India aged 50+ with diabetes (INR millions)

	Loss due to blindness (Eckert assumptions)	Loss due to blindness (Shamanna assumptions)	Loss due to MSVI (Eckert assumptions)
Lost productivity of people with sight loss aged 50-69 unable to work	138,900	111,100	0
Lost productivity of people with sight loss aged 50-69 able to work partially	0	20,800	273,800
Lost productivity of family carers for all people with blindness or MSVI	13,900	13,900	45,600
Total productivity lost	152,800	145,800	319,400

3.4 Loss of quality adjusted life years (QALYs)

We estimate that the quality of life (QoL) of a blind person is 0.395 lower than that of a person with no sight problem after standardising by age (since blind people are on average older than those without sight problems). Blindness thus means that for each year a blind person lives they enjoy 0.395 fewer QALYs than a person without sight problems. The derivation of this estimate is set out in the supplementary material. For 1.34 million blind people with diabetes aged 40 and over 0.53 million QALYs are lost per year due to blindness. Similarly we estimate that the QoL of a person with MSVI is 0.256 lower than that of a person with no sight problem,

For peer review only - http://bmjopen.bmj.com/site/about/guidelines.xhtml

after standardising for age. For 9.10 people with MSVI and diabetes aged 40+, 2.33 million QALYs are lost per year due to MSVI. If each QALY is valued at the GNI per capita for India, for example, the value of these 2.86 million lost QALYs would be INR421.4 billion per year.

As explained above, we conducted sensitivity analyses where we equated MSVI with some problems seeing and severe problems seeing categories (instead of just the former of these) and the counterfactual with no problems seeing and slight problems seeing categories (instead of just the former of these). Under these variants, the loss was in the range 2.5 million to 3.0 million QALYs per year.

3.5 Sensitivity analyses

If the average costs per person of screening for DR and eye examination where required were 10% higher (lower) than the INR 400 and INR 1,150 respectively estimated for Kerala, the overall cost of screening would be INR 46.5 billion (INR 38.0 billion) instead of INR 42.3.

If the average unit costs of treating eye conditions were 10% higher (lower) than the figures in table 5, the overall cost of treatment would be INR 3.16 billion (INR 2.58 billion) instead of INR 2.87 billion.

We examined the alternative set of assumptions used by Shamanna on lost productivity among people with diabetes who are blind, as explained above. Under those assumptions the estimated loss due to blindness would be INR 146 billion, compared with INR 153 billion than using the Eckert assumptions. However, if people with MSVI could produce 85% of the output of those with no visual impairment, as opposed to 70% assumed by Eckert, the loss of output due to blindness and MSVI would be INR 335 billion rather than INR 472 billion; but if 5% of those with MSVI could not work at all, the loss of output would be INR 536 billion.

We examined alternative assumptions on the derivation of estimated loss of QALYs from blindness and MSVI, as discussed in the supplementary material. Under those variants, the loss was in the range 2.5 million to 3.0 million QALYs per year.

4 Discussion

For peer review only - http://bmjopen.bmj.com/site/about/guidelines.xhtml

In this study we provide estimates of several types of cost that occur as a result of blindness and MSVI amongst people aged 40 and over with diabetes in India. We estimate that, if 100% of eligible people attended, annual screening followed by eye examination where required would cost around INR 42.3 billion per year, and treating sight problems amongst people with diabetes would cost INR 2.9 billion per year, if 20% of those needing treatment are treated annually. The estimated lost economic activity per year is INR 472 billion; and loss of quality of life is estimated at around 2.86 million QALYs per year. The estimate of lost production, however, is highly sensitive to the proportion of people with MSVI able to work and how their output compares with that of a person with no visual impairment.

4.1 Previous studies

There have been previous studies looking at the economic burden of diabetes, blindness and DR in countries across the world but none to our knowledge that have looked at this issue specifically in India. Also, those studies that we have identified tend to focus on specific aspects of cost which makes comparisons with our study difficult.

Toth et al (2020) [12] found that the direct cost of treating DR in Hungary was US$145.8 million in 2016 and will increase to US$169.0 million by 2045 but did not estimate lost economic activity. Work in Singapore [13] shows that, on average, people with DR utilise medical care costing almost four times as much as a similar group of diabetic patients without DR.

The total national cost of diagnosed type 2 diabetes in Iran was estimated at US $3.78 billion in 2009 [14], consisting of US $2.04 billion direct (medical and non-medical) costs and US $1.73 billion indirect costs. Ophthalmic complications made up 14% of this figure and permanent disability, of which blindness would be a major proportion, was responsible for US $452.4 million of the indirect costs. Research from Germany [15] estimated that the total societal cost of DR was €3.51 bn for the year 2002.

Shamanna (1998) [17] estimated that the burden of blindness – in terms of lost output – in India for the year 1997 was INR 159 billion (US$ 4.4 billion), which equates to INR 665 billion at 2020 prices [28]. Shamanna, however, while not covering MSVI, considered all blind people of all ages and not just blindness among people aged 40+ with diabetes.

For peer review only - http://bmjopen.bmj.com/site/about/guidelines.xhtml

There have been several studies of related topics in the US. Schmier (2009) [29] found that Medicare payments were on average $182 higher per year amongst diabetic claimants with non-proliferative retinopathy than those without ($10,613 v $9,981), and $3,825 higher amongst patients with proliferative retinopathy ($13,806).

Lee et al (2008) [16] found that amongst a sample of US employees in 2004, direct and indirect annual costs of treating employees with DR ($18,218 and $3548) were significantly higher than those without DR (direct = $11,898; indirect = $2374).

It was estimated that in 2004 [28], the direct medical costs of treating DR amongst Americans aged 40 to 64 were approximately $300 million, although more recent work suggests that this is a very small proportion of the burden caused by diabetes as a whole - the total estimated economic burden of diagnosed diabetes in the US in 2017 [30] was $327 billion, which consisted of $237 billion in direct medical costs and $90 billion in reduced productivity.

A paper relevant to this work by Orji et al (2021) [31] analysed the cost of treating people with DR at a tertiary eye care centre in South India. They considered direct costs of the consultation, investigations, intravitreal injections, and vitreoretinal surgeries. Furthermore, they included indirect costs of transportation and boarding-lodging costs (estimates from a nearby hotel). Their median cost was INR 8,214 but includes patients who received a wide spectrum of treatments.

4.2 Strengths and limitations

The major strength of this paper is that it is, to the best of our knowledge, the first to estimate the costs of DR screening and treatment, the value of economic activity lost and the value of QALYs lost due to blindness and MSVI amongst people with diabetes in India.

We have been able to utilise data on the costs of treating patients with DR from a recent new pilot scheme that has been operating in Kerala that uses accessible technology to screen diabetic patients and refer them where necessary for follow-up investigation and treatment in hospital. Whilst we use detailed recent data from Kerala, we cannot be certain that the costs of screening would be similar across other states in India.

For peer review only

Our estimate of the value of lost productivity uses recent data on the prevalence of blindness among people with diabetes and of average incomes in India. A significant limitation however is that, in the absence of evidence on the proportion of blind people with diabetes and of people with MSVI and diabetes who are unable to work, we have had to use assumptions: we used the same assumptions as Eckert et al.. We considered using data from the 2011 Census instead of those assumptions, but there was concern from experts we consulted about the plausibility of Census data, as the Census uses different definitions of blindness and sight loss. This is an area that deserves further exploration.

Our estimate relates to the current position. If screening was introduced for all people aged 40+ with diabetes, there would in due course be fewer blind people and people with MSVI with diabetes and both the QALYs lost and the economic activity lost would be lower. On the other hand, the number of people in India with diabetes is expected to grow significantly in the next decades [32], leaving more people at risk of DR.

There is a potential issue with overlap between estimated QALY loss and economic activity loss such that summing both elements would be controversial and does not seem appropriate. Caution is required when adding the estimated cost of screening and treatment to either the estimated QALY loss or economic activity loss since the costs relate to different groups of people with diabetes. The costs of screening relate to people with diabetes whether or not they have sight problems. The costs of treatment relate mainly to people who have MSVI and are at risk of blindness. The costs of lost productivity and lost QALYs relate to people who are blind or have MSVI. In practice the treatment costs that would be incurred annually would be the costs of treating those newly diagnosed following screening, which would for at least several years include part of a backlog of people who have had deteriorating sight which could have been detected earlier if there had been screening. We assume that in the early years some 10% to 20% of those needing treatment would be treated each year.

4.3 Implications

The most important findings of this study are that the annual cost to the Indian economy arising from lost productivity due to blindness and MSVI among people with diabetes is substantial, and that the cost of annual screening of people with diabetes for eye conditions and treating those with sight-threating DR, cataract or glaucoma would also be substantial. Over time, however, the costs of treatment are likely to fall, when the backlog of people with diabetes with

For peer review only - http://bmjopen.bmj.com/site/about/guidelines.xhtml

untreated eye conditions is cleared. This is before considering the impact of demographic change and inflation. Also, over time the costs incurred through lost productivity will also decline, as the proportion of people with diabetes who become blind falls due to regular screening and timely treatment of those at risk of losing their sight. A policy to conduct annual screening followed by treatment where required would therefore yield a net benefit to the Indian economy as well as a benefit to people with diabetes at risk of blindness and their families.

Contributorship statement

SR developed the original concept; acquired data; performed analysis; drafted the paper and approved the submitted version.

RA contributed to elements of the original concept; performed analysis; contributed to the drafting of the paper and approved the submitted version.

RR contributed to acquiring cost data and approved the submitted version.

SS advised on the concept; provided expert guidance throughout; acquired data; and approved the submitted version.

RW contributed to the original concept; acquired data; performed analysis; drafted parts of the paper and approved the submitted version.

# ORNATE INDIA PROJECT GROUP

Dr Bipin Gopal	Directorate of Health Services, Thiruvananthapuram, Kerala.
Mr Rajeev Sadanandan	Health Systems Transformation Platform, New Delhi.
Dr Vasudeva Iyer Sahasranamam	Regional Institute of Ophthalmology, Thiruvananthapuram, Kerala.
Dr Simon George	Regional Institute of Ophthalmology, Thiruvananthapuram, Kerala.
Professor Gopalakrishnan Netuveli	Institute for Connected Communities, University of East London, London.
Dr Ramachandran Rajalakshmi	Department of Diabetology, Ophthalmology and Epidemiology, Madras Diabetes Research Foundation & Dr. Mohan's Diabetes Specialities Centre, Chennai.
Dr Deepa Mohan	Department of Diabetology, Ophthalmology and Epidemiology, Madras Diabetes Research Foundation & Dr. Mohan's Diabetes Specialities Centre, Chennai.
Dr Viswanathan Mohan	Department of Diabetology, Ophthalmology and Epidemiology, Madras Diabetes Research Foundation & Dr. Mohan's Diabetes Specialities Centre, Chennai.

For peer review only - http://bmjopen.bmj.com/site/about/guidelines.xhtml

Dr Taraprasad Das	Anant Bajaj Retina Institute-Srimati Kanuri Santhamma Centre for Vitreoretinal diseases, Hyderabad Eye Research Foundation, LV Prasad Eye Institute, Hyderabad.
Dr Dolores Conroy	Vision Sciences, UCL Institute of Ophthalmology, London, UK.
Miss Lakshmi Premnazir	Directorate of Health Services, Thiruvananthapuram Kerala.
Ms Jyotsna Srinath	Institute for Connected Communities, University of East London, London, UK.
Dr Pramod Bhende	Sankara Nethralaya, Chennai, Tamil Nadu
Ms Janani Surya	Sankara Nethralaya, Chennai, Tamil Nadu
Mrs Radha Ramakrishnan	Vision Sciences, UCL, London
Dr Rupak Roy	Sankara Nethralaya, Kolkata
Dr Supita Das	Sankara Nethralaya, Kolkata
Dr George Manayath	Aravind Eye Hospital, Coimbatore, Tamil Nadu
Dr Vignesh T Prabhakaran	Aravind Eye Hospital, Madurai, Tamil Nadu
Dr Giridhar Anantharaman	Giridhar Eye Institute, Cochin, Kerala
Dr Mahesh Gopalakrishnan	Giridhar Eye Institute, Cochin, Kerala
Dr Sundaram Natarajan	Aditya Jyot Hospital, Mumbai, Maharashtra
Dr Radhika Krishnan	Aditya Jyot Hospital, Mumbai, Maharashtra
Dr Sheena Liz Mani	Dr Tony Fernandez Eye Hospital, Aluva, Kerala
Dr Manisha Agarwal	Dr Shroff's Charity Eye Hospital, New Delhi
Dr. Padmaja Kumari Rani	LV Prasad Eye Institute, Hyderabad, Telangana
Dr Umesh Behera	LV Prasad Eye Institute, Bhubaneshwar, Odisha
Dr Harsha Bhattacharjee	Sri Sankaradeva Nethralaya, Guwahati, Assam
Dr Manabjyoti Barman	Sri Sankaradeva Nethralaya, Guwahati, Assam
Dr Gajendra Chawla	Vision Academy–The Socio Medical Society, Bhopal, Madhya Pradesh
Dr Alok Sen	Sadguru Netra Chikitsalaya, Chitrakoot, Madhya Pradesh
Dr Moneesh Saxena	Aurobindo Nethralaya, Raipur, Chhattisgarh
Dr Asim K Sil	Netra Niramay Niketan, Haldia, West Bengal
Dr Subhratanu Chakabarty	Netra Niramay Niketan, Haldia, West Bengal
Dr Thomas Cherian	Little Flower Hospital and Research Centre, Angamaly, Kerala
Dr Reesha Jitesh	Little Flower Hospital and Research Centre, Angamaly, Kerala
Dr Rushikesh Naigaonkar	Netra Niramay Niketan, Haldia, West Bengal
Dr Abishek Desai	Netra Niramay Niketan, Haldia, West Bengal
Dr Sucheta Kulkarni	HV Desai Hospital, Pune, Maharashtra

Competing interests

None reported.

Funding

For peer review only

This work is part of the ORNATE India project funded by the GCRF UKRI (MR/P207881/1). The research is supported by the NIHR Biomedical Research Centre at Moorfields Eye Hospital NHS Foundation Trust and UCL Institute of Ophthalmology.

Data sharing statement

The data used in this study is available upon request.

Acknowledgement

The authors would like to thank the experts who contributed information relating to the cost of ophthalmology treatment in India.

Ethics approval:

This study does not involve human or animal participants and ethical approval is not required.

References

1 World Health Organization, 2019. World report on vision.

2 Pizzarello, L., Abiose, A., Ffytche, T., Duerksen, R., Thulasiraj, R., Taylor, H., Faal, H., Rao, G., Kocur, I. and Resnikoff, S., 2004. VISION 2020: The Right to Sight: a global initiative to eliminate avoidable blindness. *Archives of ophthalmology*, *122*(4), pp.615-620.

3 National Blindness and Visual Impairment Survey 2015-2019. Dr Rajendra Prasad Centre for Ophthalmic Sciences, AIIMS, New Delhi.

4 Teo, Z.L., Tham, Y.C., Yu, M., Chee, M.L., Rim, T.H., Cheung, N., Bikbov, M.M., Wang, Y.X., Tang, Y., Lu, Y. and Wong, I.Y., 2021. Global prevalence of diabetic retinopathy and projection of burden through 2045: systematic review and meta-analysis. *Ophthalmology*, *128*(11), pp.1580-1591.

5 Zheng, Y., Ley, S.H. and Hu, F.B., 2018. Global aetiology and epidemiology of type 2 diabetes mellitus and its complications. *Nature reviews endocrinology*, *14*(2), pp.88-98.

6 Larg, A. and Moss, J.R., 2011. Cost-of-illness studies. Pharmacoeconomics, 29(8), pp.653-671.

7 Sivaprasad, S., Raman, R., Conroy, D., Wittenberg, R., Rajalakshmi, R., Majeed, A., Krishnakumar, S., Prevost, T., Parameswaran, S., Turowski, P. and Maheswari, U., 2020. The ORNATE India Project: United Kingdom–India Research Collaboration to tackle visual impairment due to diabetic retinopathy. *Eye*, *34*(7), pp.1279-1286.

8 Sivaprasad, S., Netuveli, G., Wittenberg, R., Khobragade, R., Sadanandan, R., Gopal, B., Premnazir, L., Conroy, D., Srinath, J., Ramakrishnan, R. and George, S., 2021. Complex interventions to implement a diabetic retinopathy care pathway in the public health system in Kerala: the Nayanamritham study protocol. *BMJ open*, *11*(6), p.e040577.

For peer review only - http://bmjopen.bmj.com/site/about/guidelines.xhtml

9 Sivaprasad, S., Sahasranamam, V.I., George, S., Sadanandan, R., Gopal, B., Premnazir, L., Conroy, D., Srinath, J., Ramakrishnan, R., Vijayanand, S.M. and Wittenberg, R., 2021. Burden of Diabetic Retinopathy amongst People with Diabetes Attending Primary Care in Kerala: Nayanamritham Project. *Journal of Clinical Medicine*, *10*(24), p.5903.

10 Sivaprasad, S., Raman, R., Rajalakshmi, R., Mohan, V., Deepa, M., Das, T., Ramasamy, K., Prevost, A.T., Wittenberg, R., Netuveli, G. and Lingam, G., 2020. Protocol on a multicentre statistical and economic modelling study of risk-based stratified and personalised screening for diabetes and its complications in India (SMART India). *BMJ open*, *10*(12), p.e039657.

11 Rein, D.B., Zhang, P., Wirth, K.E., Lee, P.P., Hoerger, T.J., McCall, N., Klein, R., Tielsch, J.M., Vijan, S. and Saaddine, J., 2006. The economic burden of major adult visual disorders in the United States. *Archives of ophthalmology*, *124*(12), pp.1754-1760.

12 Tóth, G., Limburg, H., Szabó, D., Sándor, G.L., Nagy, Z.Z. and Németh, J., 2021. Rapid assessment of avoidable blindness-based healthcare costs of diabetic retinopathy in Hungary and its projection for the year 2045. *British Journal of Ophthalmology*, *105*(8), pp.1116-1120.

13 Zhang, X., Low, S., Kumari, N., Wang, J., Ang, K., Yeo, D., Yip, C.C., Tavintharan, S., Sum, C.F. and Lim, S.C., 2017. Direct medical cost associated with diabetic retinopathy severity in type 2 diabetes in Singapore. *PloS one*, *12*(7), p.e0180949.

14 Javanbakht, M., Baradaran, H.R., Mashayekhi, A., Haghdoost, A.A., Khamseh, M.E., Kharazmi, E. and Sadeghi, A., 2011. Cost-of-illness analysis of type 2 diabetes mellitus in Iran. *PloS one*, *6*(10), p.e26864.

15 Happich, M., Reitberger, U., Breitscheidel, L., Ulbig, M. and Watkins, J., 2008. The economic burden of diabetic retinopathy in Germany in 2002. *Graefe's Archive for Clinical and Experimental Ophthalmology*, *246*(1), pp.151-159.

16 Lee, L.J., Yu, A.P., Cahill, K.E., Oglesby, A.K., Tang, J., Qiu, Y. and Birnbaum, H.G., 2008. Direct and indirect costs among employees with diabetic retinopathy in the United States. *Current medical research and opinion*, *24*(5), pp.1549-1559.

17 Shamanna, B.R., Dandona, L. and Rao, G.N., 1998. Economic burden of blindness in India. *Indian journal of ophthalmology*, *46*(3), p.169.

18 https://www.statista.com/statistics/995968/india-diabetes-prevalence-among-males-by-age-group/, https://www.statista.com/statistics/996000/india-diabetes-prevalence-among-females-by-age-group/ (accessed October 2021)

19 https://www.populationpyramid.net/india/2020/ (accessed October 2021)

20 Steinmetz, J.D., Bourne, R.R., Briant, P.S., Flaxman, S.R., Taylor, H.R., Jonas, J.B., Abdoli, A.A., Abrha, W.A., Abualhasan, A., Abu-Gharbieh, E.G. and Adal, T.G., 2021. Causes of blindness and vision impairment in 2020 and trends over 30 years, and prevalence of avoidable blindness in relation to VISION 2020: the Right to Sight: an analysis for the Global Burden of Disease Study. The Lancet Global Health, 9(2), pp.e144-e160.

21 Wittenberg, R., Anderson, R., Redding S., Gopal, B., Sadanandan, R., Sahasranamam, V.I., George, S., Premnazir, L., Netuveli, G., Srinath, J., Ramakrishnan, R., Conroy, D. and Sivraprasad, S. 2023. Costs and cost-effectiveness of the Kerala Pilot Screening Programme for Diabetic Retinopathy in the Public Health System, medRxiv preprint.

For peer review only - http://bmjopen.bmj.com/site/about/guidelines.xhtml

22 John, D. and Parikh, R., 2017. Cost-effectiveness and cost utility of community screening for glaucoma in urban India. *Public Health*, *148*, pp.37-48.

23 Le, H.G., Ehrlich, J.R., Venkatesh, R., Srinivasan, A., Kolli, A., Haripriya, A., Ravindran, R.D., Thulasiraj, R.D., Robin, A.L., Hutton, D.W. and Stein, J.D., 2016. A sustainable model for delivering high-quality, efficient cataract surgery in southern India. *Health Affairs*, *35*(10), pp.1783-1790.

24 Eckert, K.A., Carter, M.J., Lansingh, V.C., Wilson, D.A., Furtado, J.M., Frick, K.D. and Resnikoff, S., 2015. A simple method for estimating the economic cost of productivity loss due to blindness and moderate to severe visual impairment. *Ophthalmic epidemiology*, *22*(5), pp.349-355.

25 https://data.worldbank.org/indicator/NY.GNP.PCAP.CN?locations=IN accessed February 2022.

26 Luo, N., Liu, G., Li, M., Guan, H., Jin, X. and Rand-Hendriksen, K., 2017. Estimating an EQ-5D-5L value set for China. *Value in Health*, *20*(4), pp.662-669.

27 https://www.iapb.org/learn/vision-atlas/causes-of-vision-loss/diabetic-retinopathy/ accessed March 2022.

28 https://data.worldbank.org/indicator/FP.CPI.TOTL?end=2020&locations=IN&start=1991 accessed December 2021.

29 Schmier, J.K., Covert, D.W., Lau, E.C. and Matthews, G.P., 2009. Medicare expenditures associated with diabetes and diabetic retinopathy. *Retina*, *29*(2), pp.199-206.

30 Care, D., 2018. Economic Costs of Diabetes in the US in 2017. *Diabetes Care*, *41*, p.917.

31 Orji, A., Rani, P.K., Narayanan, R., Sahoo, N.K. and Das, T., 2021. The economic burden of diabetic retinopathy care at a tertiary eye care center in South India. *Indian Journal of Ophthalmology*, *69*(3), p.666.

32 https://www.diabetesatlas.org/data/en/country/93/in.html accessed December 2021.

For peer review only

Derivation of the QALY loss associated with blindness and moderate to severe visual impairment (MSVI)

The SMART dataset has responses to questions about sight:

1. I have no problems seeing
2. I have slight problems seeing
3. I have some problems seeing
4. I have severe problems seeing
5. I am unable to see

We equate categories 4 and 5 with blindness and category 3 with MSVI. The comparator is category 1.
The dataset also has responses to the EQ-5D-5L descriptors:

Table 1: Loss of QALYs from five levels of five categories					
Level	Mobility	Self Care	Usual Activities	Pain and discomfort	Anxiety and depression
1	I have no problems in walking about	I have no problems washing or dressing myself	I have no problems with performing my usual activities	I have no pain or discomfort	I am not anxious or depressed
2	I have slight problems	I have mild problems	I have mild problems	I have mild pain or discomfort	I am mildly anxious or depressed
3	I have moderate problems	I have moderate problems	I have moderate problems	I have moderate pain or discomfort	I am moderately anxious or depressed
4	I have severe problems	I have severe problems	I have severe problems	I have severe pain or discomfort	I am severely anxious or depressed
5	I am unable to walk about	I am unable to wash or dress myself	I am unable to perform my usual activities	I have extreme pain or discomfort	I am extremely anxious or depressed

For peer review only

The Chinese value set enables the quality adjusted life-year (QALY) value to be derived for a respondent reporting any combination of these levels.

The value set is as follows:[*]

Table 2: EQ-5D-5L: loss of QALYs from five levels of five forms of health related quality of life					
Level	Mobility	Self Care	Usual Activities	Pain and discomfort	Anxiety and Depression
1	-	-	-	-	-
2	0.066	0.048	0.045	0.058	0.049
3	0.158	0.116	0.107	0.138	0.118
4	0.287	0.210	0.194	0.252	0.215
5	0.345	0.253	0.233	0.302	0.258

For example, a respondent registering level 2 on mobility, 3 on self care, 1 on usual activities, 2 on pain and discomfort and 5 on anxiety and depression would be assigned a QALY level of

1 - 0.066 - 0.116 - 0 - 0.138 - 0.258 = 0.422

We can now estimate the mean age-adjusted QALY loss with blindness and MSVI.

[*]Luo N, Liu G, Li M, Guan H, Jin X, Rand-Hendriksen K. Estimating an EQ-5D-5L value set for China. Value Health. 2017 Apr;20(4):662-669. doi: 10.1016/j.jval.2016.11.016. Epub 2017 Feb 9. Table 4 column 3.

Table 3: QALY loss with blindness and MSVI						
		Mean QALY by age			Mean QALY by age	
Age (1)	Numbers blind (2)	Blind (3)	No problems seeing (4)	Numbers MSVI (5)	MSVI (6)	No problems seeing (7)
40-44	2	0.340	0.980	10	0.805	As in column (4)
45-49	9	0.590	0.975	25	0.670	
50-54	9	0.550	0.966	54	0.711	
55-59	11	0.627	0.963	56	0.681	
60-64	15	0.442	0.948	75	0.718	
65-69	15	0.605	0.938	109	0.724	
70-74	18	0.509	0.914	92	0.653	
75-79	8	0.595	0.883	57	0.590	
80-84	10	0.355	0.828	54	0.539	
85-89	2	0.208	0.823	18	0.475	
90-94	3	0.266	0.559	6	0.483	
95-99	1	0.481	0.559	1	0.481	
	103			557		
Age weighted mean (blind or MSVI weights)		0.512	0.907		0.662	0.918

The data in columns 3 and 6 are the tariff values corresponding to the EQ5D responses of those SMART participants who report vision categories 4 and 5 or vision category 3 respectively.

QALY loss with blindness = 0.907 - 0.512 = 0.395
QALY loss with MSVI = 0.918 - 0.662 = 0.256

For peer review only

Sensitivity analysis blind =5 and half of 4 MSVI = 3 and half of 4

Table 3: QALY loss with blindness and MSVI						
		Mean QALY by age			Mean QALY by age	
Age (1)	Numbers blind (2)	Blind (3)	No problems seeing (4)	Numbers MSVI (5)	MSVI (6)	No problems seeing (7)
40-44	1.5	0.252	0.980	10.5	0.795	As in column (4)
45-49	4.5	0.590	0.975	29.5	0.658	
50-54	5	0.558	0.966	58	0.699	
55-59	5.5	0.627	0.963	61.5	0.676	
60-64	8	0.429	0.948	82	0.695	
65-69	8	0.576	0.938	116	0.718	
70-74	9.5	0.485	0.914	100.5	0.643	
75-79	4	0.595	0.883	61	0.591	
80-84	5.5	0.305	0.828	58.5	0.529	
85-89	1	0.208	0.823	19	0.461	
90-94	1.5	0.266	0.559	7.5	0.440	
95-99	0.5	0.481	0.559	1.5	0.481	
	54.5			605.5		
Age weighted mean (blind or MSVI weights)		0.492	0.913		0.652	0.918

QALY loss with blindness = 0.913 - 0.492 = 0.421
QALY loss with MSVI = 0.918 - 0.652 = 0.266

For peer review only

Sensitivity analysis

Blind = 4 and 5; MSVI = 3; Comparator = 1 and 2

Table 5: QALY loss with blindness and MSVI

		Mean QALY by age			Mean QALY by age	
Age (1)	Numbers blind (2)	Blind (3)	No problems seeing (4)	Numbers MSVI (5)	MSVI (6)	No problems seeing (7)
40-44	2	0.340	0.968	10	0.805	As in column (4)
45-49	9	0.590	0.962	25	0.670	
50-54	9	0.550	0.950	54	0.711	
55-59	11	0.627	0.938	56	0.681	
60-64	15	0.442	0.914	75	0.718	
65-69	15	0.605	0.899	109	0.724	
70-74	18	0.509	0.862	92	0.653	
75-79	8	0.595	0.832	57	0.590	
80-84	10	0.355	0.784	54	0.539	
85-89	2	0.208	0.766	18	0.475	
90-94	3	0.266	0.678	6	0.483	
95-99	1	0.481	0.639	1	0.481	
	103			557		
Age weighted mean (blind or MSVI weights)		0.512	0.882		0.662	0.883

QALY loss with blindness = 0.882 - 0.512 = 0.370

QALY loss with MSVI = 0.883 - 0.662 = 0.221

For peer review only

BMJ Open

ESTIMATING THE COSTS OF BLINDNESS AND MODERATE TO SEVERE VISUAL IMPAIRMENT AMONGST PEOPLE WITH DIABETES IN INDIA

Journal:	*BMJ Open*
Manuscript ID	bmjopen-2022-063390.R2
Article Type:	Original research
Date Submitted by the Author:	10-May-2023
Complete List of Authors:	Redding, Stuart; University of Oxford Nuffield Department of Primary Care Health Sciences, Centre for Health Service Economics & Organisation<br>Anderson, Robert; University of Oxford Nuffield Department of Primary Care Health Sciences, Centre for Health Service Economics & Organisation<br>Raman, Rajiv; Sankara Nethralaya, Shri Bhagwan Mahavir Vitreoretinal services<br>Sivaprasad, Sobha; Moorfields Eye Hospital<br>Wittenberg, Raphael; University of Oxford, Centre for Health Service Economics and Organisation
**Primary Subject Heading**:	Health economics
Secondary Subject Heading:	Ophthalmology, Diabetes and endocrinology
Keywords:	HEALTH ECONOMICS, OPHTHALMOLOGY, General diabetes < DIABETES & ENDOCRINOLOGY

SCHOLARONE™
Manuscripts

I, the Submitting Author has the right to grant and does grant on behalf of all authors of the Work (as defined in the below author licence), an exclusive licence and/or a non-exclusive licence for contributions from authors who are: i) UK Crown employees; ii) where BMJ has agreed a CC-BY licence shall apply, and/or iii) in accordance with the terms applicable for US Federal Government officers or employees acting as part of their official duties; on a worldwide, perpetual, irrevocable, royalty-free basis to BMJ Publishing Group Ltd ("BMJ") its licensees and where the relevant Journal is co-owned by BMJ to the co-owners of the Journal, to publish the Work in this journal and any other BMJ products and to exploit all rights, as set out in our licence.

The Submitting Author accepts and understands that any supply made under these terms is made by BMJ to the Submitting Author unless you are acting as an employee on behalf of your employer or a postgraduate student of an affiliated institution which is paying any applicable article publishing charge ("APC") for Open Access articles. Where the Submitting Author wishes to make the Work available on an Open Access basis (and intends to pay the relevant APC), the terms of reuse of such Open Access shall be governed by a Creative Commons licence – details of these licences and which Creative Commons licence will apply to this Work are set out in our licence referred to above.

Other than as permitted in any relevant BMJ Author's Self Archiving Policies, I confirm this Work has not been accepted for publication elsewhere, is not being considered for publication elsewhere and does not duplicate material already published. I confirm all authors consent to publication of this Work and authorise the granting of this licence.

For peer review only

ESTIMATING THE COSTS OF BLINDNESS AND MODERATE TO SEVERE VISUAL IMPAIRMENT AMONGST PEOPLE WITH DIABETES IN INDIA

Stuart Redding*[1], Robert Anderson[1], Rajiv Raman[2], Sobha Sivaprasad[3] and Raphael Wittenberg[1] on behalf of the ORNATE India project group.[#]

[1] Centre for Health Service Economics & Organisation (CHSEO), University of Oxford
[2] Shri Bhagwan Mahavir Vitreoretinal services, Sankara Nethralaya, Chennai, India
[3]NIHR Biomedical Research Centre, Moorfields Eye Hospital NHS Foundation Trust, London

* Corresponding author:
Centre for Health Service Economics & Organisation
Radcliffe Primary Care Building
Radcliffe Observatory Quarter
Woodstock Road
Oxford OX2 6GG
Email: stuart.redding@phc.ox.ac.uk

For peer review only

For peer review only - http://bmjopen.bmj.com/site/about/guidelines.xhtml

Abstract

Objectives: This study provides an estimate of the annual cost of blindness and moderate to severe visual impairment (MSVI) among people with diabetes aged 40 and above in Indian subcontinent in the year 2019.

Design: A cost of illness study

Setting: India

Participants: People with diabetes aged 40 years and above in India in the year 2019.

Primary and secondary outcome measures: We provide estimates for the total costs of screening for most common vision threatening eye conditions, treatment of these conditions, economic activity lost by these people and their family carers whose ability to work is affected, and loss of quality of life experienced by people with diabetes and blindness or MSVI.

Results: We estimate that for people with diabetes aged 40 years or above, annual screening followed by eye examination where required would cost around INR 42.3 billion (4230 crores) per year; treating sight problems around INR 2.87 billion (287 crores) per year if 20% of those needing treatment receive it; and lost economic activity around INR 472 billion (47200 crores) and that 2.86 million (0.286 crores) quality-adjusted life years (QALYs) are lost annually due to blindness and MSVI. The estimate of lost production is highly sensitive to the proportion of people with MSVI able to work and how their output compares with that of a person with no visual impairment.

Conclusions: We believe that this is the first study to estimate the cost of blindness and MSVI for people aged 40 and over with diabetes in India. The annual cost to the Indian economy is substantial. We would expect this cost to fall if a successful screening and treatment plan is introduced in India. We suggest further work using more robust data, when available, to estimate the loss of productivity and loss of QALYs would be worthwhile.

For peer review only - http://bmjopen.bmj.com/site/about/guidelines.xhtml

'Strengths and limitations of this study'

- We believe that this is the first study to estimate the cost of blindness and MSVI for people aged 40 and over with diabetes in India.
- A range of data is employed including recent data on costs of screening for diabetic retinopathy in Kerala, but it is not possible to be certain that the costs of screening would be similar across other states in India.
- We take care to make realistic assumptions, in particular that it would not be realistic for the full backlog of people needing treatment to all be treated in just one year.
- Two alternative sets of assumptions are made regarding lost productivity: collection of data would be valuable to enable a more robust estimate to be made of lost productivity.
- The data on the quality of life of people with eye conditions is drawn from a recent study in India, but the sample with severe sight problems is fairly small.

1. Introduction

Blindness, defined by the World Health Organisation as Snellen visual acuity of 3/60 or worse in the better eye [1], is a debilitating condition which, in addition to the disabling consequences for the person affected, generates substantial care and treatment costs for society. While some people who suffer from vision loss are able to work, their productivity is likely to be less than that of a person with better vision and some may drop out of the labour force entirely. Therefore blindness and sight loss reduces the potential output of the economy by decreasing the output of both those who are directly affected and those who have to stop or reduce work in order to provide unpaid care for people who are blind.

Significant work has been undertaken to measure and reduce the prevalence of blindness in India over the last twenty years. Much of this work has been influenced by or undertaken directly as part of the Vision 2020 initiative implemented by the World Health Organisation (WHO), which aimed to eliminate avoidable blindness by 2020 [2], and the WHO Global Action Plan for Universal Eye Health 2014-2019, which set a target of a reduction in the prevalence of visual impairment by 25% by 2019 from the baseline level of 2010. Actions taken have facilitated a 47.1% reduction in the prevalence of blindness, 52.6% in Moderate to Severe Visual Impairment (MSVI) and 51.9% in Visual Impairment (VI) during this time period, mainly by reducing the cataract burden and by correcting refractive errors [3].

For peer review only

One of the main preventable causes of blindness is diabetic retinopathy (DR). This is a microvascular complication of diabetes that can cause diabetic macular oedema or proliferative diabetic retinopathy, both of which can result in vision impairment and progress to irreversible sight loss. Together they are termed sight threatening DR (STDR). Among individuals with diabetes, the global prevalence of DR was 22.3% in 2020 [4]. As life expectancy continues to increase, the numbers of people with diabetes and complications such as DR are expected to rise unless people adopt healthier lifestyles or preventive interventions become available [5]. In addition, people with diabetes are also affected by other common causes of blindness such as cataract and glaucoma and so rates of blindness in people with diabetes exceeds those of people without diabetes.

This study reports the findings of a cost of illness study in which we attempt to quantify an annual value of the total economic impact of blindness and treatment of sight-threatening conditions amongst people with diabetes aged 40 years and over in India. Cost of illness studies identify and measure direct, indirect and intangible costs associated with a disease and show the total burden of that disease on society: see, for example, Larg and Moss (2011) [6] for a summary of the approaches utilised and their limitations. Our cost of illness study estimates the total costs of screening people aged 40 and over with diabetes, healthcare for those found to have eye conditions and lost productivity by those who are blind or experience MSVI and by family and friends providing unpaid care for them. We use data on the prevalence of blindness and MSVI among people with diabetes; service utilisation by those with eye conditions and associated cost data from multiple sources; and economic activity lost due to blindness or MSVI by people with diabetes and by those who stop or reduce work to care for them. An estimate of the loss in quality of life experienced by people with diabetes who have blindness or MSVI is also provided.

Monitoring and screening patients with diabetes for DR also identifies other eye conditions that cause visual impairment that require treatment. We therefore include the costs of treatment of cataract and glaucoma as well as of DR amongst people with diabetes presenting with MSVI or blindness.

This cost of illness study is part of the ORNATE-India research project [7] which aims to evaluate cost-effective measures for screening for diabetes and its complications and to examine the potential impact of a reduction in the prevalence of blindness on the Indian

For peer review only - http://bmjopen.bmj.com/site/about/guidelines.xhtml

economy. The project includes an evaluation of a pilot screening programme for DR in Kerala, the Nayanamritham project [8,9], and an evaluation of a community screening programme for diabetes and its complications in 20 areas of India covering all the six regions, the SMART India project[10].

The direct medical cost of DR alone has been explored in countries such as the USA [11], Hungary [12], and Singapore [13]. There has also been cost-of-illness analysis of Type II diabetes and its complications in lower-middle income countries (LMIC) such as Iran [14]. The economic impact of DR alone has been measured in Germany [15] and the USA [16], amongst other countries, but, to the best of our knowledge, this is the first attempt to adopt a holistic and pragmatic approach for India incorporating all common causes of blindness and MSVI that can affect people with diabetes.

Studies to measure the economic impact of all-cause blindness in India have been limited. The most recent complete estimate of the economic cost of blindness in India [17] of which we are aware was produced more than twenty years ago. That paper produced an estimate of the burden of blindness in India of INR159 billion for 1997, and a cumulative lifetime loss of INR 2,787 billion. It also estimated that treating all cases of blindness due to cataract would cost INR5.3 billion but did not evaluate the impact of STDR in people with diabetes.

The estimate provided here relates to the annual cost of retinal screening for people aged 40 and over who have diabetes in 2020, identifying and treating all common causes of preventable blindness and MSVI and lost productivity by people with diabetes who are blind or have MSVI and their unpaid carers. This is known as the prevalence approach to cost of illness studies. The incidence approach, which would provide an estimate of the lifetime costs for a cohort of people with diabetes-related blindness, is not adopted.

The costs of screening and treatment (direct costs) and economic costs of lost productivity (indirect costs) relate to different cohorts. The direct costs relate to people who are referred to hospitals for treatment after being identified during the screening programme as having eye conditions and the indirect costs relate to people who are blind or have MSVI. It is important to understand the overall annual costs incurred by the Indian economy covering both those relating to screening and treatment, including those incurred by patients, and those relating to lost productivity that would be avoided through screening and treatment. Understanding the

For peer review only - http://bmjopen.bmj.com/site/about/guidelines.xhtml

overall economic consequences of blindness is crucial to ensure that the case for devoting resources to the prevention, treatment and research on eye disease is understood and justified.

2. Research methods

2.1 Overall Approach

We sought to estimate societal costs of blindness and MSVI amongst people with diabetes aged 40 years and over in India in 2019, including costs of screening and healthcare and of lost economic output for blind people with diabetes, people with MSVI and diabetes and their carers. Results for these three categories of costs are provided separately. All reported estimates are annual costs for India, in INR at 2019 prices (although some of the prevalence data relate to 2020). Cost estimates represent a snapshot for the year and not lifetime costs. Discounting was unnecessary since all costs refer to a 1-year period.

2.2 Data sources

Estimates were derived using data from multiple sources as discussed below.

2.2.1 Number of people affected

We begin by defining the number of people aged 40 and over who have diabetes and people who are 50 and over who have diabetes and blindness or MSVI due to existing eye conditions. Age specific prevalence data for diabetes are drawn from the Global Burden of Disease study [18] and combined with 2020 population data from www.PopulationPyramid.net [19] to provide an estimated aggregate number of people in India aged 40 and over with diabetes in the year 2020. A recent study by the Global Burden of Disease (GBD) Study [20] showed prevalence rates for a number of health conditions for South Asia. As most of the population in South Asia is from India, it is appropriate to use these values to estimate the number of cases in India. The age standardised prevalence of blindness in people aged 50 years or above in South Asia is 29.8/1000.

The Global Burden of Disease 2020 prevalence rates per 1,000 population aged 50 and over for common eye conditions among people with blindness and people with MSVI are set out in Table 1. Also shown are estimates of the number of people (in thousands) aged 50 and over

For peer review only - http://bmjopen.bmj.com/site/about/guidelines.xhtml

with diabetes and each of the eye conditions. These estimates assume that, with the exception of DR which can only affect people with diabetes, the prevalence of each eye condition is the same for people with diabetes as for the wider population. The prevalence in the table, which relate to total population aged 50 and over, imply DR rates of 3.07 among people with diabetes who are blind and 7.86 among people with diabetes who have MSVI. Summing across the eye conditions implies that there are around 1,335,000 blind people aged 50 and over with diabetes and 9,096,000 people with MSVI and diabetes aged 50 and over.

Table 1: Numbers of people with diabetes in India suffering from blindness or MSVI by eye conditions

	Number of people (50+)	Aged 50+ crude prevalence (per 1,000 people)		
With diabetes in India	41,194,000	153.86		
	**Number of people with diabetes and blindness (50+)**	**Aged 50+ crude prevalence (per 1,000 people)**	**Number of people with diabetes and MSVI (50+)**	**Aged 50+ crude prevalence (per 1,000 people)**
All causes	1,335,000	29.80	9,096,000	33.98
Due to cataract	758,000	18.40	3,489,200	84.70
Due to glaucoma	74,000	1.79	121,900	2.96
Due to age related macular degeneration	38,000	0.92	156,100	3.79
Due to STDR	126,000	0.47	324,000	1.21
Due to uncorrected refractive error	125,200	3.04	4,119,500	100.00
Due to residual causes	214,000	5.20	885,700	21.50

2.2.2 Costing care

Blindness can be treated in some conditions such as cataract but not in others including advanced age-related macular degeneration and diabetic retinopathy. However, there are

For peer review only - http://bmjopen.bmj.com/site/about/guidelines.xhtml

treatments available to slow the progression and/or prevent blindness and MSVI due to most of these common conditions. We attempt to measure the aggregate costs of these treatments in India. The costs of care are based on evidence from previous literature, expert advice and calculations derived from a pilot scheme run to screen for DR among people with diabetes in Kerala [10] which was conducted as part of the ORNATE-India research project described in the introduction.

Data from the Nayanamritham study in Kerala suggests a unit cost of initial screening of approximately INR 400, if all people with diabetes in the non-communicable disease (NCD) register are screened. This estimate includes INR 85 staff costs, INR 25 cost of admin support, INR 200 annuitized cost of the cameras and 105 annuitized cost of staff training in the Kerala pilot. Information on staff salaries, staff time per person screened, costs of cameras and costs of staff training were provided by the Kerala team. Further details are reported elsewhere (Wittenberg et al, 2023) [21].

Furthermore, in this study 31% of screened patients were referred to hospital services. These may be due to cataract, glaucoma, STDR or ungradable retinal images. The estimated unit cost of these referrals was INR 1,150 comprising INR 200 staff costs of the eye examination and INR 950 travel and lost income costs to the patient and an attendant.

We also consider the cost of treating those who were found through the screening programme to have sight-threatening conditions. The unit costs, including costs to the patient and their family, are estimated as an average across private providers and health insurance coverage. These include INR 15,000 for cataract surgery, INR 5,000 for glaucoma surgery [22] and INR 12,000 for DR treatment (laser or intravitreal injections) [23]. The unit costs and sources of our estimates for these interventions are presented in table 2 below. The expert advice was provided by senior clinicians from the SMART India research project.

For peer review only - http://bmjopen.bmj.com/site/about/guidelines.xhtml

Table 2: Unit cost of treatment (INR)

Item	Health care	Travel	Total cost	Source
Screening	400	0	400	Kerala pilot
Eye examination	200	950	1150	Kerala pilot
Glasses	1000		1000	Expert advice
Eye drops	500		500	Expert advice
Cataract surgery	15000	950	15950	Expert advice
STDR treatment	12000	2850	14850	Expert advice
Glaucoma surgery	5000	950	5950	John et al 2017
Glaucoma average	1625	238	1862.5	Expert advice
Age related macular degeneration	12000	2850	14850	Expert advice

Cost of blindness estimates for high income countries take into account the cost of low vision rehabilitation, community care and residential care for people suffering from blindness. However, Since these services are in their infancy in India and most patients rely on family carers, costs are not included for them. Costs for hip replacement or depression, which are also included in the costs of blindness in high income countries, have been omitted from the calculations presented here.

2.2.3 Costing economic losses

The indirect economic costs of blindness and MSVI accrue from loss of output due to reduced productivity of blind or visually impaired people compared to people with full vision, plus the loss of output by carers who leave employment or reduce their working hours to look after family members or friends who are blind.

For peer review only - http://bmjopen.bmj.com/site/about/guidelines.xhtml

For peer review only

Determining an estimate for the aggregate loss of output in India requires use of data relating to hours of work and productivity lost by blind and visually impaired people who could be economically active if they had full sight and hours of work lost by carers who have to cease or reduce work in order to care for blind friends or family members. Some of these data are available and others need to be estimated. Where data are not available, we use the same assumptions as those adopted by Eckert et al (2015) [24]. In their study, they assume that no blind people are able to work, and that for each blind person one potentially active worker forgoes 10% of their productive time to care for the blind person. Furthermore, they assume that all people with MSVI are able to work, but only produce 70% of what a person with full sight produces. They assume that for each person with MSVI one potentially active worker forgoes 5% of their productive time to care for the person with MSVI.

Furthermore, people with sight loss over 70 are assumed to be economically inactive regardless of their vision. For the purpose of our cost estimate full-time equivalent output per person is valued at the Indian average Gross National Income (GNI)) per capita. This was INR147,524 in 2019 [25]. With these assumptions, economic activity lost (EAL in the equation below) can be calculated as:

EAL (blind) = (number blind under 70 + total number blind x 10%) x (GNP per capita)

EAL (MSVI) = (number MSVI under 70 x 30% + total number MSVI x 5%) x (GNP per capita)

As a sensitivity analysis, an estimate is presented for blind people based on assumptions used by Shamanna et al (1998) [17] in their study of all-cause blindness in India. The assumptions taken from Shamanna are as follows:

a) 80% of blind people are economically inactive
b) the average blind person who is economically active produces 25% of the output achieved by a worker with full sight
c) for each blind person, one economically active worker forgoes 10% of their productive time to care for the blind person.

They do not provide estimates for people with MSVI.

For peer review only - http://bmjopen.bmj.com/site/about/guidelines.xhtml

For peer review only

2.2.4 Calculating QALY losses

Ill-health can affect health-related quality of life in many ways. The standard metric for measuring health-related quality of life in cost-utility analyses is the quality adjusted life year (QALY). One QALY is equal to one life year 1 in full health; any ill-health that reduces ability to perform day-to-day functions is assumed to reduce quality of life, such that one life year in less than full health is valued at less than one QALY. The standard QALY metric is now increasingly EQ-5D-5L, replacing EQ-5D-3L. Each respondent indicates on a five-point scale their ability to function in five specific dimensions (mobility, self-care, usual activities, pain/discomfort, and anxiety/depression). Each EQ-5D-5L health state can be assigned a value on a scale or tariff where 1=full health and 0=dead. Since there is currently no value set available for India, the Chinese value set [26] is used in this study.

In order to quantify the QALY losses associated with blindness and with MSVI, we use individual-level data on these five dimensions combined with data detailing the state of their sight. This data is taken from the SMART study, which was part of the overall ORNATE India study as outlined above. The SMART study collected information on EQ-5D-5L health state and on vision using the following self-reported categories:

1. I have no problems seeing
2. I have slight problems seeing
3. I have some problems seeing
4. I have severe problems seeing
5. I am unable to see

For the purpose of our analysis, blindness is defined as responses in the categories "I have severe problems seeing" or "I am unable to see" and MSVI when the respondent said "I have some problems seeing".

To estimate the loss of QALYs due to blindness and MSVI the response category "I have no problems seeing" is treated as the counterfactual against which to estimate the loss. The Chinese value set [26] was applied to the EQ5D data for each person to get a quality of life (QoL) value for each person. Since the aim is to derive the average reduced quality of life due to poor sight in poor sight it is necessary to establish the average QoL those with poor sight

For peer review only - http://bmjopen.bmj.com/site/about/guidelines.xhtml

would have had if they had good sight. Since their average age is greater than those with good sight, and QoL level falls with age, the age distribution of those with poor sight is applied to the age specific QoL values in those with good sight to derive the appropriate comparator – the average QALY if those with poor sight had good sight. Further details of the methods employed in this analysis are presented in the supplementary material.

2.2.5 Sensitivity analyses

Sensitivity analysis was performed on three aspects of the analysis.

We estimate costs based on 10% higher (lower) cost per person for DR screening and eye examination, and also if the average unit costs of treating eye conditions were 10% higher (lower) than the figures in Table 2.

The set of assumptions used by Shamanna et al [17] on lost productivity among people with diabetes who are blind was considered, as explained above, as an alternative to the Eckert et al [24] assumptions. .

Alternative assumptions on the derivation of estimated loss of QALYs from blindness and MSVI were examined as presented in the supplementary material. In this sensitivity analysis we continue to equate blindness with "I am unable to see" and MSVI with "I have some problems seeing" but share the "I have severe problems seeing" category equally between those who are blind and those who have MSVI. A second definition of the counterfactual was also employed in which the "I have slight problems seeing" category was added to the "I have no problems seeing" group.

2.2.6 Patient and Public Involvement

Patients and the public were not involved in this study.

3. Results

3.1 Prevalence of blindness amongst people with diabetes

This study considered people resident in India aged 40 and over who have diabetes. Based on age-stratified prevalence data from the Global Burden of Disease (GBD) for 2019 and age-

For peer review only - http://bmjopen.bmj.com/site/about/guidelines.xhtml

stratified population data from PopulationPyramid.net for 2020, we estimate that there were 55.9 million people aged 40 and over and almost 41.2 million people aged 50 and over in India with diabetes in 2020. As presented in Table 1 above, using GBD data it is estimated that, of these 41.2 million people aged 40 and over, there are some 1.34 million blind people with diabetes and 9.10 million people with MSVI and diabetes. The breakdown of these estimates by eye condition causing blindness or MSVI is also shown in Table 1.

3.2 Cost of screening and follow-up treatment

We assume that annual retinal screening will be offered to all 55.9 million people with diabetes aged 40 and over. The annual cost of screening this group would be INR 22.3 billion assuming the cost of retinal screening per person is INR 400 on the basis of the Kerala pilot.

On the basis of the Kerala pilot screening programme in which 31% of those screened were referred for an eye examination, an estimated 17.3 million people of the 55.9 million people screened would be referred to hospital services for an eye examination. If all of these attended hospital for an eye examination at a cost of INR 1,150 per individual, the cost of eye examinations for this group would be INR 19.9 billion. The estimated total cost of annual screening followed by eye examination where required would therefore be around INR 42.3 billion. This data is presented in table 3 below.

Table 3: Cost of screening for sight problems amongst people with diabetes aged 40+

	**Annual**	**Cost (INR millions)**		
Item	Number (millions)	Health care	Travel	Total cost
Screening	55.87	22,350		22,350
Eye examination	17.32	3,460	16,450	19,920
Total		25,810	16,450	42,270
Note: 1) All people with diabetes aged 40+ are assumed to be screened<br>2) 31% of those screened are assumed to need eye examination, as per Kerala pilot				

We also consider the cost of treating those found to have DR, cataract or glaucoma and the cost of glasses for those with refractive disorders, using the prevalence rates and unit costs of treatment shown above. Treatment for AMD or for people with other eye conditions is not

For peer review only

For peer review only - http://bmjopen.bmj.com/site/about/guidelines.xhtml

considered. Only people aged 50 and over are considered since the GBD does not present data for people aged 40 to 49. Since the prevalence of blindness and MSVI in this group are likely to be low, the omission of this group is unlikely to have a large impact on the numbers of people treated.

It seems improbable that all those with prevalent eye conditions could be treated in one year: it is likely to require many years for the backlog to be cleared. On the basis that 10% could be treated each year, the cost would be 1.43 billion INR per year, including 110 million INR for patient and attendant travel and loss of income. On the basis that 20% of the backlog could be treated each year, the cost would be 2.87 billion INR per year, including 220 million INR for patient and attendant travel and loss of income (Table 4). Almost 73% of this cost relates to cataract surgery.

Table 4: Cost of treating sight problems amongst diabetics aged 50+ in India, if 20% of those needing treatment are treated each year

Intervention	Annual	Cost (INR millions)		
	Number (thousands)	Health care	Travel	Total cost
Cataract surgery	151.60	2,270	140	2,420
STDR treatment	25.27	300	70	380
Glaucoma surgery	3.69	20	4	20
Glaucoma eye drops	14.75	7	-	7
Glasses	42.84	40	-	40
**Total**	238.15	2,650	220	2,870

3.3 Economic activity lost

We also estimate the value of economic activity lost due to blindness and MSVI amongst people with diabetes who are blind or have MSVI, following the method described in section 2.2.3.

Our estimates are of the basis of:

a) Economic activity lost due to blindness using Eckert et al (2015) [24] assumptions
b) Economic activity lost due to blindness using Shamanna et al (1998) [17] assumptions
c) Economic activity lost due to MSVI using Eckert assumptions

For peer review only - http://bmjopen.bmj.com/site/about/guidelines.xhtml

It is assumed that output is lost only in the case of people aged 50 to 69 and that those over 70 would not work even if they had better vision. Data are used on the numbers of people who experience blindness and MSVI caused by DR, by age, from South-East Asia [26], since we could not find data by age for all people with diabetes and blindness or MSVI in India. In this data, 29.5% blind people aged 50+ with DR are over 70 and 32.0% of those with MSVI aged 50+ with DR are over 70. Therefore economic activity relating to 941,000 blind people and 6.186 billion people with MSVI is considered. We use average gross national income (GNI) per capita as an indicator of the value of lost production. GNI per capita was INR 147,524 in 2019 [25].

The economic loss due to blindness is estimated as INR 153 billion using the Eckert assumptions or INR 146 billion under the Shamanna assumptions, and the economic loss due to MSVI is estimated at INR 319 billion under the Eckert assumptions (Table 5).

Table 5: Economic loss as a consequence of blindness or MSVI among the population of India aged 50+ with diabetes (INR millions)

	Loss due to blindness (Eckert assumptions)	Loss due to blindness (Shamanna assumptions)	Loss due to MSVI (Eckert assumptions)
Lost productivity of people with sight loss aged 50-69 unable to work	138,900	111,100	0
Lost productivity of people with sight loss aged 50-69 able to work partially	0	20,800	273,800
Lost productivity of family carers for all people with blindness or MSVI	13,900	13,900	45,600

For peer review only - http://bmjopen.bmj.com/site/about/guidelines.xhtml

Total productivity lost	152,800	145,800	319,400

3.4 Loss of quality adjusted life years (QALYs)

The quality of life (QoL) of a blind person is estimated to be 0.395 lower than that of a person with no sight problem after standardising by age (since blind people are on average older than those without sight problems). Blindness thus means that for each year a blind person lives they enjoy 0.395 fewer QALYs than a person without sight problems. The derivation of this estimate is set out in the supplementary material. For 1.34 million blind people with diabetes aged 40 and over, 0.53 million QALYs are lost per year due to blindness. Similarly we estimate that the QoL of a person with MSVI is 0.256 lower than that of a person with no sight problem, after standardising for age. For 9.10 million people with MSVI and diabetes aged 40+, 2.33 million QALYs are lost per year due to MSVI.

3.5 Sensitivity analyses

If the average costs per person of screening for DR and eye examination where required were 10% higher (lower) than the INR 400 and INR 1,150 respectively estimated for Kerala, the overall cost of screening and eye examinations would be INR 46.5 billion (INR 38.0 billion) instead of INR 42.3.

If the average unit costs of treating eye conditions were 10% higher (lower) than the figures in table 5, the overall cost of treatment would be INR 3.16 billion (INR 2.58 billion) instead of INR 2.87 billion.

The alternative set of assumptions used by Shamanna on lost productivity among people with diabetes who are blind, was also considered, as explained above. Under those assumptions the estimated loss due to blindness would be INR 146 billion, compared with INR 153 billion using the Eckert assumptions. However, if people with MSVI could produce 85% of the output of those with no visual impairment, as opposed to 70% assumed by Eckert, the loss of output due

For peer review only - http://bmjopen.bmj.com/site/about/guidelines.xhtml

to blindness and MSVI would be INR 335 billion rather than INR 472 billion; but if 5% of those with MSVI could not work at all, the loss of output would be INR 536 billion.

Additionally, alternative assumptions on the derivation of estimated loss of QALYs from blindness and MSVI, were examined as discussed in the supplementary material. Under those variants, the loss was in the range of 2.5 million to 3.0 million QALYs per year.

4 Discussion

In this study we provide estimates of several types of cost that occur as a result of blindness and MSVI amongst people aged 40 and over with diabetes in India. If 100% of eligible people attended, annual screening followed by eye examination where required would cost around INR 42.3 billion per year, and treating sight problems amongst people with diabetes would cost INR 2.9 billion per year, if 20% of those needing treatment are treated annually. The estimated lost economic activity per year is INR 472 billion; and loss of quality of life is estimated at around 2.86 million QALYs per year. The estimate of lost production, however, is highly sensitive to the proportion of people with MSVI able to work and how their output compares with that of a person with no visual impairment.

4.1 Previous studies

There have been previous studies looking at the economic burden of diabetes, blindness and DR in countries across the world but none to our knowledge that have looked at this issue specifically in India. Also, those studies identified tend to focus on specific aspects of cost which makes comparisons with our study difficult.

Toth et al (2020) [12] found that the direct cost of treating DR in Hungary was US$145.8 million in 2016 and will increase to US$169.0 million by 2045 but did not estimate lost economic activity. Work in Singapore [13] shows that, on average, people with DR utilise medical care costing almost four times as much as a similar group of diabetic patients without DR.

The total national cost of diagnosed type 2 diabetes in Iran was estimated at US $3.78 billion in 2009 [14], consisting of US $2.04 billion direct (medical and non-medical) costs and US $1.73 billion indirect costs. Ophthalmic complications made up 14% of this figure and permanent disability, of which blindness would be a major proportion, was responsible for US

For peer review only - http://bmjopen.bmj.com/site/about/guidelines.xhtml

$452.4 million of the indirect costs. Research from Germany [15] estimated that the total societal cost of DR was €3.51 billion for the year 2002.

Shamanna (1998) [17] estimated that the burden of blindness – in terms of lost output – in India for the year 1997 was INR 159 billion (US$ 4.4 billion), which equates to INR 665 billion at 2020 prices [27]. Shamanna, however, while not covering MSVI, considered all blind people of all ages and not just blindness among people aged 40+ with diabetes.

There have been several studies of related topics in the US. Schmier (2009) [28] found that Medicare payments were on average $182 higher per year amongst diabetic claimants with non-proliferative retinopathy than those without ($10,613 v $9,981), and $3,825 higher amongst patients with proliferative retinopathy ($13,806).

Lee et al (2008) [16] found that amongst a sample of US employees in 2004, direct and indirect annual costs of treating employees with DR ($18,218 and $3548) were significantly higher than those without DR (direct = $11,898; indirect = $2374).

It was estimated that in 2004 [11], the direct medical costs of treating DR amongst Americans aged 40 to 64 were approximately $300 million, although more recent work suggests that this is a very small proportion of the burden caused by diabetes as a whole - the total estimated economic burden of diagnosed diabetes in the US in 2017 [29] was $327 billion, which consisted of $237 billion in direct medical costs and $90 billion in reduced productivity.

A paper relevant to this work by Orji et al (2021) [30] analysed the cost of treating people with DR at a tertiary eye care centre in South India. They considered direct costs of the consultation, investigations, intravitreal injections, and vitreoretinal surgeries. Furthermore, they included indirect costs of transportation and boarding-lodging costs (estimates from a nearby hotel). Their median cost was INR 8,214 but includes patients who received a wide spectrum of treatments.

4.2 Strengths and limitations

The major strength of this paper is that it is, to the best of our knowledge, the first to estimate the costs of DR screening and treatment, the value of economic activity lost and the value of QALYs lost due to blindness and MSVI amongst people with diabetes in India.

For peer review only

We have been able to utilise data on the costs of treating patients with DR from a recent new pilot scheme that has been operating in Kerala that uses accessible technology to screen diabetic patients and refer them where necessary for follow-up investigation and treatment in hospital. Whilst detailed recent data from Kerala is used, we cannot be certain that the costs of screening would be similar across other states in India.

Our estimate of the value of lost productivity uses recent data on the prevalence of blindness among people with diabetes and of average incomes in India. A significant limitation however is that, in the absence of evidence on the proportion of blind people with diabetes and of people with MSVI and diabetes who are unable to work, assumptions have been required. The same assumptions as Eckert et al were employed. An alternative considered was to use data from the 2011 Census instead of those assumptions, but there was concern from experts about the plausibility of Census data, as the Census uses different definitions of blindness and sight loss. This is an area that deserves further exploration.

Our estimate relates to the current position. If screening was introduced for all people aged 40+ with diabetes, there would in due course be fewer blind people and people with MSVI with diabetes and both the QALYs lost and the economic activity lost would be lower. On the other hand, the number of people in India with diabetes is expected to grow significantly in the next decades [31], leaving more people at risk of DR.

There is a potential issue with overlap between estimated QALY loss and economic activity loss such that summing both elements would be controversial and does not seem appropriate. Caution is required when adding the estimated cost of screening and treatment to either the estimated QALY loss or economic activity loss since the costs relate to different groups of people with diabetes. The costs of screening relate to people with diabetes whether or not they have sight problems. The costs of treatment relate mainly to people who have MSVI and are at risk of blindness. The costs of lost productivity and lost QALYs relate to people who are blind or have MSVI. In practice the treatment costs that would be incurred annually would be the costs of treating those newly diagnosed following screening, which would for at least several years include part of a backlog of people who have had deteriorating sight which could have been detected earlier if there had been screening. We assume that in the early years some 10% to 20% of those needing treatment would be treated each year.

For peer review only

4.3 Implications

The most important findings of this study are that the annual cost to the Indian economy arising from lost productivity due to blindness and MSVI among people with diabetes is substantial, and that the cost of annual screening of people with diabetes for eye conditions and treating those with sight-threating DR, cataract or glaucoma would also be substantial. Over time, however, the costs of treatment are likely to fall, when the backlog of people with diabetes with untreated eye conditions is cleared. This is before considering the impact of demographic change and inflation. Also, over time the costs incurred through lost productivity will also decline, as the proportion of people with diabetes who become blind falls due to regular screening and timely treatment of those at risk of losing their sight. A policy to conduct annual screening followed by treatment where required would therefore yield a net benefit to the Indian economy as well as a benefit to people with diabetes at risk of blindness and their families.

Contributorship statement

SR developed the original concept; acquired data; performed analysis; drafted the paper and approved the submitted version.

RA contributed to elements of the original concept; performed analysis; contributed to the drafting of the paper and approved the submitted version.

RR contributed to acquiring cost data and approved the submitted version.

SS advised on the concept; provided expert guidance throughout; acquired data; and approved the submitted version.

RW contributed to the original concept; acquired data; performed analysis; drafted parts of the paper and approved the submitted version.

# ORNATE INDIA PROJECT GROUP

Dr Bipin Gopal	Directorate of Health Services, Thiruvananthapuram, Kerala.
Mr Rajeev Sadanandan	Health Systems Transformation Platform, New Delhi.
Dr Vasudeva Iyer Sahasranamam	Regional Institute of Ophthalmology, Thiruvananthapuram, Kerala.
Dr Simon George	Regional Institute of Ophthalmology, Thiruvananthapuram, Kerala.
Professor Gopalakrishnan Netuveli	Institute for Connected Communities, University of East London, London.

For peer review only - http://bmjopen.bmj.com/site/about/guidelines.xhtml

Dr Ramachandran Rajalakshmi	Department of Diabetology, Ophthalmology and Epidemiology, Madras Diabetes Research Foundation & Dr. Mohan's Diabetes Specialities Centre, Chennai.
Dr Deepa Mohan	Department of Diabetology, Ophthalmology and Epidemiology, Madras Diabetes Research Foundation & Dr. Mohan's Diabetes Specialities Centre, Chennai.
Dr Viswanathan Mohan	Department of Diabetology, Ophthalmology and Epidemiology, Madras Diabetes Research Foundation & Dr. Mohan's Diabetes Specialities Centre, Chennai.
Dr Taraprasad Das	Anant Bajaj Retina Institute-Srimati Kanuri Santhamma Centre for Vitreoretinal diseases, Hyderabad Eye Research Foundation, LV Prasad Eye Institute, Hyderabad.
Dr Dolores Conroy	Vision Sciences, UCL Institute of Ophthalmology, London, UK.
Miss Lakshmi Premnazir	Directorate of Health Services, Thiruvananthapuram Kerala.
Ms Jyotsna Srinath	Institute for Connected Communities, University of East London, London, UK.
Dr Pramod Bhende	Sankara Nethralaya, Chennai, Tamil Nadu
Ms Janani Surya	Sankara Nethralaya, Chennai, Tamil Nadu
Mrs Radha Ramakrishnan	Vision Sciences, UCL, London
Dr Rupak Roy	Sankara Nethralaya, Kolkata
Dr Supita Das	Sankara Nethralaya, Kolkata
Dr George Manayath	Aravind Eye Hospital, Coimbatore, Tamil Nadu
Dr Vignesh T Prabhakaran	Aravind Eye Hospital, Madurai, Tamil Nadu
Dr Giridhar Anantharaman	Giridhar Eye Institute, Cochin, Kerala
Dr Mahesh Gopalakrishnan	Giridhar Eye Institute, Cochin, Kerala
Dr Sundaram Natarajan	Aditya Jyot Hospital, Mumbai, Maharashtra
Dr Radhika Krishnan	Aditya Jyot Hospital, Mumbai, Maharashtra
Dr Sheena Liz Mani	Dr Tony Fernandez Eye Hospital, Aluva, Kerala
Dr Manisha Agarwal	Dr Shroff's Charity Eye Hospital, New Delhi
Dr. Padmaja Kumari Rani	LV Prasad Eye Institute, Hyderabad, Telangana
Dr Umesh Behera	LV Prasad Eye Institute, Bhubaneshwar, Odisha
Dr Harsha Bhattacharjee	Sri Sankaradeva Nethralaya, Guwahati, Assam
Dr Manabjyoti Barman	Sri Sankaradeva Nethralaya, Guwahati, Assam
Dr Gajendra Chawla	Vision Academy–The Socio Medical Society, Bhopal, Madhya Pradesh
Dr Alok Sen	Sadguru Netra Chikitsalaya, Chitrakoot, Madhya Pradesh
Dr Moneesh Saxena	Aurobindo Nethralaya, Raipur, Chhattisgarh
Dr Asim K Sil	Netra Niramay Niketan, Haldia, West Bengal
Dr Subhratanu Chakabarty	Netra Niramay Niketan, Haldia, West Bengal
Dr Thomas Cherian	Little Flower Hospital and Research Centre, Angamaly, Kerala
Dr Reesha Jitesh	Little Flower Hospital and Research Centre, Angamaly, Kerala
Dr Rushikesh Naigaonkar	Netra Niramay Niketan, Haldia, West Bengal
Dr Abishek Desai	Netra Niramay Niketan, Haldia, West Bengal
Dr Sucheta Kulkarni	HV Desai Hospital, Pune, Maharashtra

For peer review only - http://bmjopen.bmj.com/site/about/guidelines.xhtml

Competing interests

None reported.

Funding

This work is part of the ORNATE India project funded by the Global Challenges Research Fund (GCRF) UK Research and Innovation (UKRI) (MR/P207881/1). The research is supported by the NIHR Biomedical Research Centre at Moorfields Eye Hospital NHS Foundation Trust and UCL Institute of Ophthalmology.

Data sharing statement

The data used in this study is available upon request.

Acknowledgement

The authors would like to thank the experts who contributed information relating to the cost of ophthalmology treatment in India.

Ethics approval:

This study does not involve human or animal participants and ethical approval is not required.

References

1 World Health Organization, 2019. World report on vision.

2 Pizzarello, L., Abiose, A., Ffytche, T., Duerksen, R., Thulasiraj, R., Taylor, H., Faal, H., Rao, G., Kocur, I. and Resnikoff, S., 2004. VISION 2020: The Right to Sight: a global initiative to eliminate avoidable blindness. *Archives of ophthalmology*, *122*(4), pp.615-620.

3 National Blindness and Visual Impairment Survey 2015-2019. Dr Rajendra Prasad Centre for Ophthalmic Sciences, AIIMS, New Delhi.

4 Teo, Z.L., Tham, Y.C., Yu, M., Chee, M.L., Rim, T.H., Cheung, N., Bikbov, M.M., Wang, Y.X., Tang, Y., Lu, Y. and Wong, I.Y., 2021. Global prevalence of diabetic retinopathy and projection of burden through 2045: systematic review and meta-analysis. *Ophthalmology*, *128*(11), pp.1580-1591.

5 Zheng, Y., Ley, S.H. and Hu, F.B., 2018. Global aetiology and epidemiology of type 2 diabetes mellitus and its complications. *Nature reviews endocrinology*, *14*(2), pp.88-98.

6 Larg, A. and Moss, J.R., 2011. Cost-of-illness studies. Pharmacoeconomics, 29(8), pp.653-671.

For peer review only - http://bmjopen.bmj.com/site/about/guidelines.xhtml

7 Sivaprasad, S., Raman, R., Conroy, D., Wittenberg, R., Rajalakshmi, R., Majeed, A., Krishnakumar, S., Prevost, T., Parameswaran, S., Turowski, P. and Maheswari, U., 2020. The ORNATE India Project: United Kingdom–India Research Collaboration to tackle visual impairment due to diabetic retinopathy. *Eye*, *34*(7), pp.1279-1286.

8 Sivaprasad, S., Netuveli, G., Wittenberg, R., Khobragade, R., Sadanandan, R., Gopal, B., Premnazir, L., Conroy, D., Srinath, J., Ramakrishnan, R. and George, S., 2021. Complex interventions to implement a diabetic retinopathy care pathway in the public health system in Kerala: the Nayanamritham study protocol. *BMJ open*, *11*(6), p.e040577.

9 Sivaprasad, S., Sahasranamam, V.I., George, S., Sadanandan, R., Gopal, B., Premnazir, L., Conroy, D., Srinath, J., Ramakrishnan, R., Vijayanand, S.M. and Wittenberg, R., 2021. Burden of Diabetic Retinopathy amongst People with Diabetes Attending Primary Care in Kerala: Nayanamritham Project. *Journal of Clinical Medicine*, *10*(24), p.5903.

10 Sivaprasad, S., Raman, R., Rajalakshmi, R., Mohan, V., Deepa, M., Das, T., Ramasamy, K., Prevost, A.T., Wittenberg, R., Netuveli, G. and Lingam, G., 2020. Protocol on a multicentre statistical and economic modelling study of risk-based stratified and personalised screening for diabetes and its complications in India (SMART India). *BMJ open*, *10*(12), p.e039657.

11 Rein, D.B., Zhang, P., Wirth, K.E., Lee, P.P., Hoerger, T.J., McCall, N., Klein, R., Tielsch, J.M., Vijan, S. and Saaddine, J., 2006. The economic burden of major adult visual disorders in the United States. *Archives of ophthalmology*, *124*(12), pp.1754-1760.

12 Tóth, G., Limburg, H., Szabó, D., Sándor, G.L., Nagy, Z.Z. and Németh, J., 2021. Rapid assessment of avoidable blindness-based healthcare costs of diabetic retinopathy in Hungary and its projection for the year 2045. *British Journal of Ophthalmology*, *105*(8), pp.1116-1120.

13 Zhang, X., Low, S., Kumari, N., Wang, J., Ang, K., Yeo, D., Yip, C.C., Tavintharan, S., Sum, C.F. and Lim, S.C., 2017. Direct medical cost associated with diabetic retinopathy severity in type 2 diabetes in Singapore. *PloS one*, *12*(7), p.e0180949.

14 Javanbakht, M., Baradaran, H.R., Mashayekhi, A., Haghdoost, A.A., Khamseh, M.E., Kharazmi, E. and Sadeghi, A., 2011. Cost-of-illness analysis of type 2 diabetes mellitus in Iran. *PloS one*, *6*(10), p.e26864.

15 Happich, M., Reitberger, U., Breitscheidel, L., Ulbig, M. and Watkins, J., 2008. The economic burden of diabetic retinopathy in Germany in 2002. *Graefe's Archive for Clinical and Experimental Ophthalmology*, *246*(1), pp.151-159.

16 Lee, L.J., Yu, A.P., Cahill, K.E., Oglesby, A.K., Tang, J., Qiu, Y. and Birnbaum, H.G., 2008. Direct and indirect costs among employees with diabetic retinopathy in the United States. *Current medical research and opinion*, *24*(5), pp.1549-1559.

17 Shamanna, B.R., Dandona, L. and Rao, G.N., 1998. Economic burden of blindness in India. *Indian journal of ophthalmology*, *46*(3), p.169.

18 https://www.statista.com/statistics/995968/india-diabetes-prevalence-among-males-by-age-group/, https://www.statista.com/statistics/996000/india-diabetes-prevalence-among-females-by-age-group/ (accessed October 2021)

19 https://www.populationpyramid.net/india/2020/ (accessed October 2021)

20 Steinmetz, J.D., Bourne, R.R., Briant, P.S., Flaxman, S.R., Taylor, H.R., Jonas, J.B., Abdoli, A.A., Abrha, W.A., Abualhasan, A., Abu-Gharbieh, E.G. and Adal, T.G., 2021. Causes of blindness and

For peer review only - http://bmjopen.bmj.com/site/about/guidelines.xhtml

vision impairment in 2020 and trends over 30 years, and prevalence of avoidable blindness in relation to VISION 2020: the Right to Sight: an analysis for the Global Burden of Disease Study. The Lancet Global Health, 9(2), pp.e144-e160.

21 Wittenberg, R., Anderson, R., Redding S., Gopal, B., Sadanandan, R., Sahasranamam, V.I., George, S., Premnazir, L., Netuveli, G., Srinath, J., Ramakrishnan, R., Conroy, D. and Sivraprasad, S. 2023. Costs and cost-effectiveness of the Kerala Pilot Screening Programme for Diabetic Retinopathy in the Public Health System, medRxiv preprint.

22 John, D. and Parikh, R., 2017. Cost-effectiveness and cost utility of community screening for glaucoma in urban India. *Public Health*, *148*, pp.37-48.

23 Le, H.G., Ehrlich, J.R., Venkatesh, R., Srinivasan, A., Kolli, A., Haripriya, A., Ravindran, R.D., Thulasiraj, R.D., Robin, A.L., Hutton, D.W. and Stein, J.D., 2016. A sustainable model for delivering high-quality, efficient cataract surgery in southern India. *Health Affairs*, *35*(10), pp.1783-1790.

24 Eckert, K.A., Carter, M.J., Lansingh, V.C., Wilson, D.A., Furtado, J.M., Frick, K.D. and Resnikoff, S., 2015. A simple method for estimating the economic cost of productivity loss due to blindness and moderate to severe visual impairment. *Ophthalmic epidemiology*, *22*(5), pp.349-355.

25 https://data.worldbank.org/indicator/NY.GNP.PCAP.CN?locations=IN accessed February 2022.

26 Luo, N., Liu, G., Li, M., Guan, H., Jin, X. and Rand-Hendriksen, K., 2017. Estimating an EQ-5D-5L value set for China. *Value in Health*, *20*(4), pp.662-669.

27 https://data.worldbank.org/indicator/FP.CPI.TOTL?end=2020&locations=IN&start=1991 accessed December 2021.

28 Schmier, J.K., Covert, D.W., Lau, E.C. and Matthews, G.P., 2009. Medicare expenditures associated with diabetes and diabetic retinopathy. *Retina*, *29*(2), pp.199-206.

29 Care, D., 2018. Economic Costs of Diabetes in the US in 2017. *Diabetes Care*, *41*, p.917.

30 Orji, A., Rani, P.K., Narayanan, R., Sahoo, N.K. and Das, T., 2021. The economic burden of diabetic retinopathy care at a tertiary eye care center in South India. *Indian Journal of Ophthalmology*, *69*(3), p.666.

31 https://www.diabetesatlas.org/data/en/country/93/in.html accessed December 2021.

For peer review only

For peer review only - http://bmjopen.bmj.com/site/about/guidelines.xhtml

Derivation of the QALY loss associated with blindness and moderate to severe visual impairment (MSVI)

The SMART dataset has responses to questions about sight:

1. I have no problems seeing
2. I have slight problems seeing
3. I have some problems seeing
4. I have severe problems seeing
5. I am unable to see

We equate categories 4 and 5 with blindness and category 3 with MSVI. The comparator is category 1.
The dataset also has responses to the EQ-5D-5L descriptors:

Table 1: Loss of QALYs from five levels of five categories

Level	Mobility	Self Care	Usual Activities	Pain and discomfort	Anxiety and depression
1	I have no problems in walking about	I have no problems washing or dressing myself	I have no problems with performing my usual activities	I have no pain or discomfort	I am not anxious or depressed
2	I have slight problems	I have mild problems	I have mild problems	I have mild pain or discomfort	I am mildly anxious or depressed
3	I have moderate problems	I have moderate problems	I have moderate problems	I have moderate pain or discomfort	I am moderately anxious or depressed
4	I have severe problems	I have severe problems	I have severe problems	I have severe pain or discomfort	I am severely anxious or depressed
5	I am unable to walk about	I am unable to wash or dress myself	I am unable to perform my usual activities	I have extreme pain or discomfort	I am extremely anxious or depressed

For peer review only

The Chinese value set enables the quality adjusted life-year (QALY) value to be derived for a respondent reporting any combination of these levels.

The value set is as follows:*

Table 2: EQ-5D-5L: loss of QALYs from five levels of five forms of health related quality of life

Level	Mobility	Self Care	Usual Activities	Pain and discomfort	Anxiety and Depression
1	-	-	-	-	-
2	0.066	0.048	0.045	0.058	0.049
3	0.158	0.116	0.107	0.138	0.118
4	0.287	0.210	0.194	0.252	0.215
5	0.345	0.253	0.233	0.302	0.258

For example, a respondent registering level 2 on mobility, 3 on self care, 1 on usual activities, 2 on pain and discomfort and 5 on anxiety and depression would be assigned a QALY level of

1 - 0.066 - 0.116 - 0 - 0.138 - 0.258 = 0.422

We can now estimate the mean age-adjusted QALY loss with blindness and MSVI.

*Luo N, Liu G, Li M, Guan H, Jin X, Rand-Hendriksen K. Estimating an EQ-5D-5L value set for China. Value Health. 2017 Apr;20(4):662-669. doi: 10.1016/j.jval.2016.11.016. Epub 2017 Feb 9. Table 4 column 3.

For peer review only

Table 3: QALY loss with blindness and MSVI						
		Mean QALY by age			Mean QALY by age	
Age (1)	Numbers blind (2)	Blind (3)	No problems seeing (4)	Numbers MSVI (5)	MSVI (6)	No problems seeing (7)
40-44	2	0.340	0.980	10	0.805	As in column (4)
45-49	9	0.590	0.975	25	0.670	
50-54	9	0.550	0.966	54	0.711	
55-59	11	0.627	0.963	56	0.681	
60-64	15	0.442	0.948	75	0.718	
65-69	15	0.605	0.938	109	0.724	
70-74	18	0.509	0.914	92	0.653	
75-79	8	0.595	0.883	57	0.590	
80-84	10	0.355	0.828	54	0.539	
85-89	2	0.208	0.823	18	0.475	
90-94	3	0.266	0.559	6	0.483	
95-99	1	0.481	0.559	1	0.481	
	103			557		
Age weighted mean (blind or MSVI weights)		0.512	0.907		0.662	0.918

The data in columns 3 and 6 are the tariff values corresponding to the EQ5D responses of those SMART participants who report vision categories 4 and 5 or vision category 3 respectively.

QALY loss with blindness = 0.907 - 0.512 = 0.395
QALY loss with MSVI = 0.918 - 0.662 = 0.256

For peer review only

Sensitivity analysis blind =5 and half of 4 MSVI = 3 and half of 4

Table 3: QALY loss with blindness and MSVI

		Mean QALY by age			Mean QALY by age	
Age (1)	Numbers blind (2)	Blind (3)	No problems seeing (4)	Numbers MSVI (5)	MSVI (6)	No problems seeing (7)
40-44	1.5	0.252	0.980	10.5	0.795	As in column (4)
45-49	4.5	0.590	0.975	29.5	0.658	
50-54	5	0.558	0.966	58	0.699	
55-59	5.5	0.627	0.963	61.5	0.676	
60-64	8	0.429	0.948	82	0.695	
65-69	8	0.576	0.938	116	0.718	
70-74	9.5	0.485	0.914	100.5	0.643	
75-79	4	0.595	0.883	61	0.591	
80-84	5.5	0.305	0.828	58.5	0.529	
85-89	1	0.208	0.823	19	0.461	
90-94	1.5	0.266	0.559	7.5	0.440	
95-99	0.5	0.481	0.559	1.5	0.481	
	54.5			605.5		
Age weighted mean (blind or MSVI weights)		0.492	0.913		0.652	0.918

QALY loss with blindness = 0.913 - 0.492 = 0.421
QALY loss with MSVI = 0.918 - 0.652 = 0.266

For peer review only

Sensitivity analysis

Blind = 4 and 5; MSVI = 3; Comparator = 1 and 2

Table 5: QALY loss with blindness and MSVI						
		Mean QALY by age			Mean QALY by age	
Age (1)	Numbers blind (2)	Blind (3)	No problems seeing (4)	Numbers MSVI (5)	MSVI (6)	No problems seeing (7)
40-44	2	0.340	0.968	10	0.805	As in column (4)
45-49	9	0.590	0.962	25	0.670	
50-54	9	0.550	0.950	54	0.711	
55-59	11	0.627	0.938	56	0.681	
60-64	15	0.442	0.914	75	0.718	
65-69	15	0.605	0.899	109	0.724	
70-74	18	0.509	0.862	92	0.653	
75-79	8	0.595	0.832	57	0.590	
80-84	10	0.355	0.784	54	0.539	
85-89	2	0.208	0.766	18	0.475	
90-94	3	0.266	0.678	6	0.483	
95-99	1	0.481	0.639	1	0.481	
	103			557		
Age weighted mean (blind or MSVI weights)		0.512	0.882		0.662	0.883

QALY loss with blindness = 0.882 - 0.512 = 0.370

QALY loss with MSVI = 0.883 - 0.662 = 0.221

For peer review only
